# Fluorinated ether electrolyte with controlled solvation structure for high voltage lithium metal batteries

**DOI:** 10.1038/s41467-022-29199-3

**Published:** 2022-05-06

**Authors:** Yan Zhao, Tianhong Zhou, Timur Ashirov, Mario El Kazzi, Claudia Cancellieri, Lars P. H. Jeurgens, Jang Wook Choi, Ali Coskun

**Affiliations:** 1 Department of Chemistry, University of Fribourg, Chemin de Musee 9, Fribourg, 1700 Switzerland; 2 Electrochemistry Laboratory, Paul Scherrer Institut, Villigen, 5232 Switzerland; 3 Laboratory for Joining Technologies and Corrosion, Swiss Federal Laboratories for Materials Science and Technology, Empa, Überlandstrasse 129, Dübendorf, CH 8600 Switzerland; 4 School of Chemical and Biological Engineering, Department of materials science and engineering, and Institute of Chemical Processes, Seoul National University, 1 Gwanak-ro, Gwanak-gu, Seoul, 08826 Republic of Korea

**Keywords:** Batteries, Batteries

## Abstract

The development of new solvents is imperative in lithium metal batteries due to the incompatibility of conventional carbonate and narrow electrochemical windows of ether-based electrolytes. Whereas the fluorinated ethers showed improved electrochemical stabilities, they can hardly solvate lithium ions. Thus, the challenge in electrolyte chemistry is to combine the high voltage stability of fluorinated ethers with high lithium ion solvation ability of ethers in a single molecule. Herein, we report a new solvent, 2,2-dimethoxy-4-(trifluoromethyl)-1,3-dioxolane (DTDL), combining a cyclic fluorinated ether with a linear ether segment to simultaneously achieve high voltage stability and tune lithium ion solvation ability and structure. High oxidation stability up to 5.5 V, large lithium ion transference number of 0.75 and stable Coulombic efficiency of 99.2% after 500 cycles proved the potential of DTDL in high-voltage lithium metal batteries. Furthermore, 20 μm thick lithium paired LiNi_0.8_Co_0.1_Mn_0.1_O_2_ full cell incorporating 2 M LiFSI-DTDL electrolyte retained 84% of the original capacity after 200 cycles at 0.5 C.

## Abstract (web summary)

The development of lithium-metal batteries is limited by the low thermodynamic and/or low voltage stability of conventional electrolytes. Here, the authors combined the high voltage stability of fluorinated ethers with high Li^+^ solvation ability of ethers in a single molecule and realized highly stable lithium-metal batteries.

## Introduction

With the increasing-demand in high density energy storage systems, the research on high-voltage (>4.0 V *vs*. Li^+^/Li) lithium (Li) metal batteries (LMBs) surged rapidly in recent years^1–4^. Li metal, as the ideal anode, provides a high theoretical specific capacity of 3860 mAh g^−1^ and a low reduction potential (–3.04 V *vs*. standard hydrogen electrode)^5–7^. When paired with a high-voltage Ni-rich cathode, the energy density of LMBs promisingly becomes almost two-times (400–500 Wh/kg) compared to that of conventional graphite-based Li-ion batteries (LIBs)^8^. The application of LMBs, however, is plagued by the thermodynamic instability of metallic Li. The uncontrollable side reactions between Li metal and electrolyte result in the formation of a fragile solid electrolyte interphase (SEI) and the mechanical failure of the SEI layer during cycling. This phenomenon causes constant consumption of both electrolyte and Li, which are accompanied by the Li dendrite growth and “dead” Li formation, thus leading to a poor cycle life^3,9,10^. Therefore, tuning the electrolyte chemistry to modulate its reactivity, electrochemical stability, ion-transport and solvation ability is a promising strategy to stabilize SEI layer and extend the cycle life^11,12^.

Commercial carbonate electrolytes have been extensively used in graphite anodes owing to their ability to form a stable SEI layer. In the case of Li metal anode (LMA), however, low concentration carbonate electrolytes (i.e., 1 M salt) usually suffer from serious side reactions, which are linked to whisker shape Li dendrite growth and significantly short cycle life. On the contrary, ether-based electrolytes tend to form relatively large and flat Li grains, which decrease the contact surface of LMA with electrolyte and exhibits high Coulombic efficiency (CE)^13,14^. However, ethers such as 1,2-dimethoxyethane (DME) and 1,3-dioxolane (DOL) are unstable in the high voltage range (i.e., >4V *vs*. Li^+^/Li) because of their poor oxidative stability at a typical salt concentration of 1 M, which naturally limits their application in high-voltage LMBs^11^. Recently, high concentration electrolytes (HCEs) with special solvent-in-salt structures reveal their compatibility with both high-voltage cathodes and LMA. The coordination of solvent molecules to the Li^+^ ions changes the solvation structure, in which the solvation sheaths become dominated by anions. Moreover, the decreased highest occupied molecular orbital-lowest unoccupied molecular orbital (HOMO-LUMO) energies of solvent molecules through coordination results in the prior decomposition of anions at low potentials, which facilitate the formation of an anion-derived inorganic SEI layer^15,16^. The high cost and viscosity of HCEs, however, limit their practical application. Recently, hydrofluoroethers (HFEs) were introduced as inert diluents. Whereas they can hardly dissolve salts and have no ionic conductivity, they offer high anodic stability and can retain similar solvent-in-salt solvation structure at low salt concentrations, which is called localized high-concentration electrolytes (LHCEs). HFEs such as tris(2,2,2-trifluoroethyl)orthoformate (TFEO), bis(2,2,2-trifluoroethyl) ether (BTFE) and 1,1,2,2-tetrafluoroethyl-2,2,3,3-tetrafluoropropylether (TTE) were widely used in high voltage LMBs.^2,17,18^. Since the HFEs need to be paired with solvents in order to compensate for the loss of ionic conductivity, the associated parasitic reactions of these solvents cannot be stopped. Moreover, there are only few reports on fluorinated ethers as solvents, which can effectively solvate Li^+^^1,11^. For example, Bao et al. elegantly showed an improved oxidative stability by lengthening alky chain and fluorination between two -O- atoms in a linear ether, and the solvation ability of fluorinated ether was maintained due to the simultaneous binding of -F and -O- atoms to Li^+^^1^. Accordingly, it is essential to design high voltage fluorinated ethers, which can effectively combine the redox stability of HFEs with the Li^+^ solvation ability and good ionic conductivity of ether-based electrolytes, while maintaining solvent-in-salt solvation cluster at normal salt concentrations. In this direction, one direct approach would be the covalent attachment of fluorinated and ether segments in order to combine all the desirable properties of electrolytes in a single molecule. The spatial arrangement of these functional groups as well as the availability of binding sites for Li^+^ are important factors in the resulting ionic conductivity, solvation ability, and redox stability of the electrolyte.

**Fig. 1:**
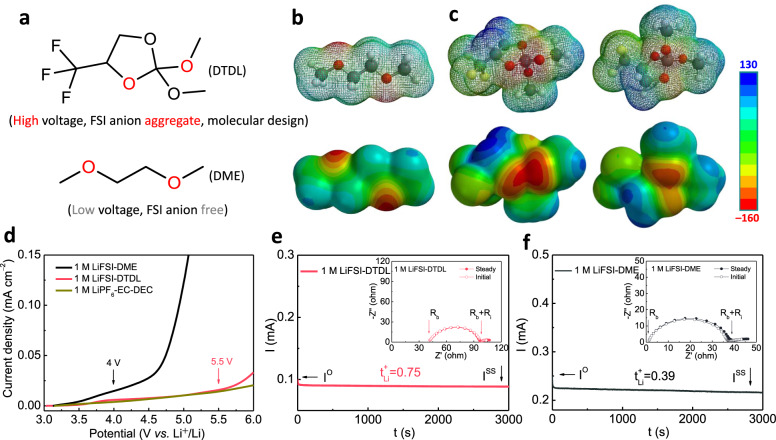
Molecular structures and electrochemical characterization. **a** The comparison of molecular structures of DTDL and DME. Electrostatic potential maps (ESP) of DME (**b**) and DTDL with front and back views (**c**), color scheme: yellow, F; red, O; gray, C; white, H. **d** Oxidation stability of three electrolytes in Li|Al half cells tested by linear sweep voltammetry (LSV). Li^+^ transference numbers and the chronoamperometry profiles of Li|Li symmetrical cells in 1 M LiFSI-DTDL (**e**) and 1 M LiFSI-DME (**f**) electrolytes under a polarization voltage of 10 mV. Insets in (**e**) and (**f**): the corresponding electrochemical impedance spectra (EIS) before and after polarization.

In this work, we present a new fluorinated ether, 2,2-dimethoxy-4-(trifluoromethyl)-1,3-dioxolane (DTDL, Fig. 1a), incorporating both cyclic fluorinated and linear ether segments. From the molecular design viewpoint, we introduced an electron-withdrawing functional group, -CF_3_, to increase oxidative stability of ether. However, in order to retain Li^+^ solvation ability, spatial arrangement of -CF_3_ was tuned to avoid direct attachment of fluorinated carbon atom to the -O- atom as in fluorinated ethers such as TFEO, BTFE, and TTE^2,17,18^. Accordingly, tetramethyl orthocarbonate core was integrated to combine cyclic fluorinated and linear ether segments in a facile, one-pot reaction to form highly preorganized binding sites with a controlled solvating ability towards Li^+^ for increased ion pairing, while simultaneously achieving high oxidative stability. After adding only 1 M of lithium bis(fluorosulfonyl)imide (LiFSI) salt, the formation of contact-ion-pair (CIP) and aggregate (AGG) clusters were observed in the DTDL-based electrolytes consistent with the previous reports^19–23^. Notably, the formation of Li^+^ coordinated FSI anion aggregate at low concentrations is quite significant as this phenomenon is usually obtained in HCEs and LHCEs. The achieved solvation structure allows an increased oxidation stability up to 5.5 V *vs*. Li/Li^+^, along with FSI-derived inorganic SEI layer and a high Li ion transference number of 0.75. Based on these exceptional features, the Li|Cu half-cell with 1 M LiFSI-DTDL exhibited a high average CE of 99.2% over 500 cycles. Moreover, the limited-excess Li|LiNi_0.8_Co_0.1_Mn_0.1_O_2_ (NCM811) full cell achieved 84% capacity retention at 0.5 C with 2 M LiFSI-DTDL electrolyte after 200 cycles, thus demonstrating the potential of this new class of electrolyte.

## Results

### Electrolyte characterization

DTDL was synthesized in a one-pot reaction through acid-catalyzed condensation of tetramethyl orthocarbonate (TMOC)^24^ and 1,1,1-trifluoro-2,3-propanediol (TFPD)^25^. The successful formation of DTDL was verified by nuclear magnetic resonance (NMR) spectroscopy analysis (Supplementary Figs. 1–8). As shown in Fig. 1a, the presence of cyclic fluorinated and linear ether segments in DTDL guaranteed high polarity and liquid state to solvate Li salt and conduct Li^+^ ions^26^ owing to the distant nature of -CF_3_ group with respect to the -O- atoms and available ether groups to maintain its solvation ability^1,11^. Electrostatic potential (ESP) maps in Fig. 1b, c revealed possible binding sites for Li^+^^1^; the negative zones on DME centered on O atoms, but DTDL showed different degrees of negative zones on different O atoms. The structure of DTDL enabled a highly preorganized binding site for Li^+^ located between the -O- atom in the cycle and the -O- atom in the linear ether segment (marked as red oxygen in Fig. 1a). Notably, the decreased electron density of -O- atom in the cyclic ether influenced by CF_3_ group resulted in a decreased Li^+^ solvation ability and altered the solvation sheath. HOMO and LUMO energy values of DTDL and DME (Supplementary Fig. 9) were calculated using density functional theory (DFT) calculations conducted at B3LYP/ 6-311++G**. Compared to DME, lower HOMO value (–7.99 eV) of DTDL exhibited the possibility of higher theoretical oxidative stability arising from electron-withdrawing -CF_3_ group, whereas the lower LUMO value (–0.26 eV) of DTDL could lead to a prior reduction on LMA. Both DTDL and DME were used to solvate 1 M LiFSI (referred to as 1 M LiFSI-DTDL and 1 M LiFSI-DME), high voltage tolerance of 1 M LiFSI-DTDL and 1 M LiFSI-DME was probed by linear sweep voltammetry (LSV) in Li|Al half cells starting from open-circuit voltage (OCV) up to 6 V at a scan rate of 5 mV s^–1^ (Fig. 1d). Unlike 1 M LiFSI-DME, which started to decompose at 4.0 V, 1 M LiFSI-DTDL exhibited higher oxidation stability up to 5.5 V. The oxidation stability of 1 M LiFSI-DTDL was also evaluated in the presence of conductive additive and binder on Al counter electrode by LSV at a scan rate of 1 mV s^−1^ and potentiostatic polarization test (Supplementary Fig. 10). This result fitted well with its lower HOMO value and proved the impact of electrolyte chemistry to control the oxidative stability. In addition, cyclic voltammetry (CV) of Li|stainless steel (SS) and Li|Al half-cells with 1 M LiFSI-DTDL also supported this result (Supplementary Figs. 11 and 12). Ionic conductivities of 1 M LiFSI-DTDL and 1 M LiFSI-DME electrolytes at different temperatures were measured (In Supplementary Fig. 13) in SS|SS blocking cell by electrochemical impedance spectroscopy (EIS) and were found to be 1.4 mS cm^–1^ and 21.3 mS cm^–1^ at 30 °C, respectively. The viscosity of DTDL, 1 M LiFSI-DTDL, DME and 1 M LiFSI-DME at 25 °C were measured to be 2.4, 8.0, 0.4, and 1.2 cP, respectively. The lithium ion transference numbers (${{{{{{\rm{t}}}}}}}_{{{{{{\rm{Li}}}}}}}^{+}$) of 1 M LiFSI-DTDL and 1 M LiFSI-DME electrolytes were recorded on the Li|Li symmetric cells (Fig. 1e, f)^27^, with the corresponding values being 0.75 and 0.39, respectively. Notably, higher ${{{{{{\rm{t}}}}}}}_{{{{{{\rm{Li}}}}}}}^{+}$ value is linked to a longer time to the onset of Li dendrite initiation based on the Sand’s time formula^28^, thus 1 M LiFSI-DTDL electrolyte is expected to show better Li dendrite suppression compared to 1 M LiFSI-DME. Wettability of electrolyte is an important factor to achieve uniform distribution of Li ion flux and consequently SEI layer^29^. Contact angle measurements of 1 M LiFSI-DTDL and 1 M LiFSI-DME electrolytes on copper (Cu) foil and separator were conducted as shown in Supplementary Fig. 14. On the Cu foil, 1 M LiFSI-DTDL exhibited a smaller contact angle of 9^o^ compared to 1 M LiFSI-DME of 17^o^. On the other hand, similar values on the separator were observed for these two electrolytes, 19^o^ from 1 M LiFSI-DTDL and 21^o^ from 1 M LiFSI-DME. The property comparisons of solvents and electrolytes are summarized in the Supplementary Table 1.

### Understanding Li plating morphology

**Fig. 2:**
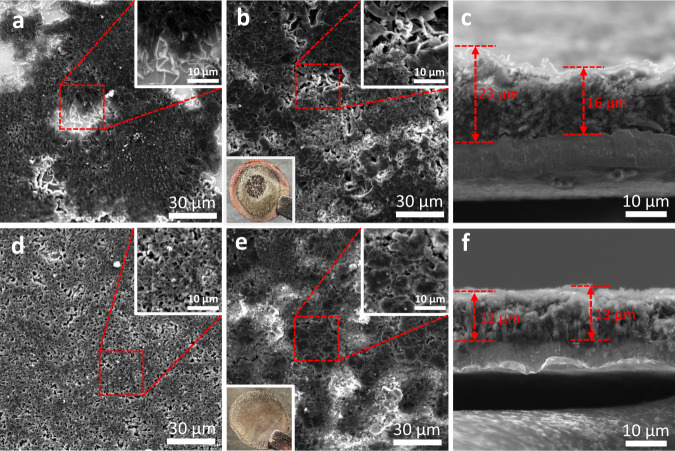
Li plating morphology on Cu foil in Li|Cu half cells. SEM images of the Li plating morphology after the first cycle in 1 M LiFSI-DME (**a**) and 1 M LiFSI-DTDL (**d**) electrolytes, insets are zoom-in images of highlighted regions. SEM images and optical photographs of the Li plated electrodes after 20 cycles in 1 M LiFSI-DME (**b**) and 1 M LiFSI-DTDL (**e**) electrolytes, insets are optical and zoom-in images. Cross-sectional SEM images of plated Li after 20 cycles in 1 M LiFSI-DME (**c**) and 1 M LiFSI-DTDL (**f**) electrolytes. All Li|Cu half cells were cycled at 1 mA cm^−2^ with a cutoff capacity of 1 mAh cm^−2^.

Li plating morphology on Cu foil in Li|Cu half cells with 1 M LiFSI-DME and 1 M LiFSI-DTDL electrolytes was observed (Fig. 2 and Supplementary Fig. 15) by scanning electron microscopy (SEM). After the first Li plating (1 mAh cm^−2^) on Cu foil at the current density of 1 mA cm^−2^, it became clear that the plated Li cannot cover the surface of Cu foil uniformly leaving significant amount of bare Cu in 1 M LiFSI-DME electrolyte and also showing slim Li whiskers at the edge (Fig. 2a), ascribed to the poor wettability of 1 M LiFSI-DME electrolyte on Cu foil. In contrast, the plated Li in 1 M LiFSI-DTDL revealed a dense coverage without any dendrites and any bare surface (Fig. 2d). The average Li grain sizes in 1 M LiFSI-DME, 1 M LiFSI-DTDL and 2 M LiFSI-DTDL were 1.32, 3.54 and 5.05 μm, respectively (Supplementary Fig. 16). Transmission electron microscopy (TEM) low-dose bright-field images of plated Li revealed the formation of a thinner (17-18 nm) and more homogeneous SEI layer in 2 M LiFSI-DTDL compared to that of 1 M LiFSI-DTDL electrolyte with the thickness of 21-27 nm (Supplementary Fig. 17). Afterwards, Li|Cu half cells were cycled for 20 cycles with different electrolytes at 1 mA cm^−2^ with a cut-off capacity of 1 mAh cm^−2^. In Fig. 2b, plated Li after cycling in 1 M LiFSI-DME showed more cracks and high surface Li filaments, which accelerate the side reactions between Li metal and electrolyte, and the optical image of Cu surface showed motley Li, peeling off of ‘dead’ Li in the central part and bare Cu surface. In stark contrast, dense morphology and bulky Li grain were observed with 1 M LiFSI-DTDL, along with uniform coverage on the Cu surface as verified by the optical image (inset, Fig. 2e). The cross-sectional SEM images revealed (Fig. 2c, f) the thicknesses of plated Li in two electrolytes. 1 M LiFSI-DTDL showed a thin and dense Li layer of 12-13 μm compared to the loose and uneven Li (16-23 μm) in the case of 1 M LiFSI-DME after 20 cycles. The evolution of the porous structure in the plated Li was also correlated with the electrochemical impedance spectroscopy (EIS) data (Supplementary Figs. 18 and 19 and Supplementary Table 2). The obvious fluctuation of charge-transfer resistance (*R*_ct_) in 1 M LiFSI-DME during cycling was attributed to the porosity increase for the decreased *R*_ct_ in the early cycles and the subsequent ‘dead’ Li formation for the increased *R*_ct_ owing to reduced active surface area, compared to stable Li plating in 1 M LiFSI-DTDL. Li plating morphology difference after cycling demonstrated clearly the advantage of DTDL over DME.

### Electrochemical characterization

**Fig. 3:**
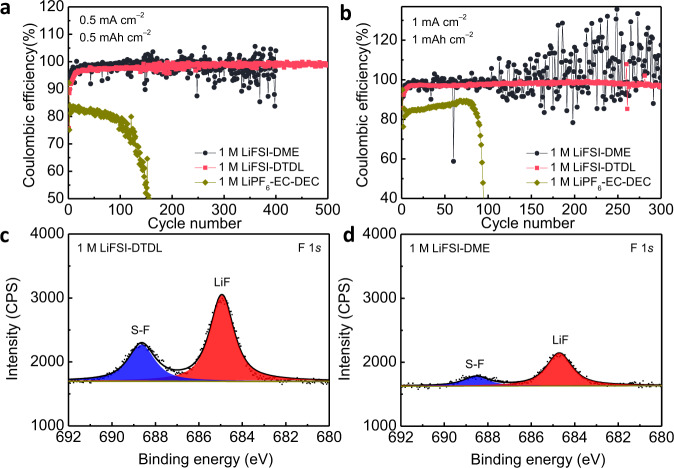
Cycling performance and SEI compositions of Li|Cu half cells. Cycling stability of Li|Cu half cells using different electrolytes at 0.5 mA cm^−2^ with a cutoff capacity of 0.5 mAh cm^−2^ (**a**) and 1 mA cm^−2^ with a cutoff capacity of 1 mAh cm^−2^ (**b**). F 1*s* XPS profiles on plated Li metal surface in Li|Cu half cells in 1 M LiFSI-DTDL (**c**) and 1 M LiFSI-DME (**d**) electrolytes after 5 cycles at 1 mA cm^−2^ with a cutoff capacity of 1 mAh cm^−2^.

In order to evaluate LMA performance, Li|Cu half cells were assembled and CEs in the presence of 1 M LiFSI-DME and 1 M LiFSI-DTDL electrolytes were measured (Fig. 3a, b and Supplementary Fig. 20). Cells with 1 M LiFSI-DME showed a clear fluctuation throughout the cycling at both current densities of 0.5 mA cm^−2^ and 1 mA cm^−2^ with the capacities of 0.5 mAh cm^−2^ and 1 mAh cm^−2^, respectively, which reflected the unstable SEI formation and irreversible plating on the LMA surface. CE values above 100% in 1 M LiFSI-DME electrolyte originated from the “dead” Li formation and its electrical reconnection, and micro short-circuit through dendrite formation during charging (Supplementary Fig. 21). On the contrary, cells with 1 M LiFSI-DTDL exhibited stable CEs over 500 cycles at 0.5 mA cm^−2^, 0.5 mAh cm^−2^ and 300 cycles at 1 mA cm^−2^, 1 mAh cm^−2^; after the stabilization of the SEI layer, average CE values reached to 99.2% (0.5 mA cm^−2^_,_ 0.5 mAh cm^−2^) and 98.2% (1 mA cm^−2^_,_ 1 mAh cm^−2^) at 250^th^ and 100^th^ cycles, respectively. Furthermore, even with a large amount of Li plating of 3 mAh cm^−2^, we still observed a stable and high average CE of 99.0% after 30 cycles with 1 M LiFSI-DTDL electrolyte (Supplementary Fig. 20). Galvanostatic cycling experiments of Li|Li symmetric cells with these electrolytes were conducted to complement the asymmetric cell measurements (Supplementary Figs. 22 and 23). The voltage hysteresis suddenly increased after 620 h in the case of 1 M LiFSI-DME, which was attributed to the severe electrolyte consumption along with repeated SEI formation during cycling. In contrast, stable polarization was observed for 1 M LiFSI-DTDL for 700 h owing to the uniform Li plating/stripping and moderate ionic conductivity^1^. EIS of the Li|Cu half cells were conducted to assess the anode-electrolyte interfacial stability (Supplementary Fig. 24). The charge transfer resistance increased clearly after 50 cycles in the cell with 1 M LiFSI-DME due to high interfacial impedance for Li^+^ ion migration^30^, whereas the impedance in the cell with 1 M LiFSI-DTDL was stable after the same number of cycles. Interface stability was also evaluated by Li|Li symmetrical cells at different cycling times with 1 M LiFSI-DME and 1 M LiFSI-DTDL electrolytes (Supplementary Fig. 25 and Supplementary Table 3), which showed a stable interface resistance for 1 M LiFSI-DTDL after a short cycling time. All the electrochemical results above proved the superiority of DTDL over DME with regard to LMA stability.

X-ray photoelectron spectroscopy (XPS) analysis was conducted on plated Li to further understand the difference in SEI composition in the Li|Cu half cells for different electrolytes (Fig. 3c, d). The F 1*s* XPS spectra revealed two identical chemical states of F in the SEI (positioned at 685 and 688.6 eV) for both electrolytes, which were assigned to Li-F and S-F bonds originating from FSI anion decomposition^31^. However, the SEI layer on Li metal surface in the 1 M LiFSI-DTDL electrolyte exhibited considerably higher LiF and S-F compositions compared to the 1 M LiFSI-DME. In addition, SEI layer was also found to be rich in N and S contents (Supplementary Figs. 26 and 27), which are typically reflective of an anion-derived SEI layer. It should be noted that the inorganic SEI layer with higher LiF content yields stable and high CE by its superior mechanical properties and electronically insulating nature^2,17,32^.

**Fig. 4:**
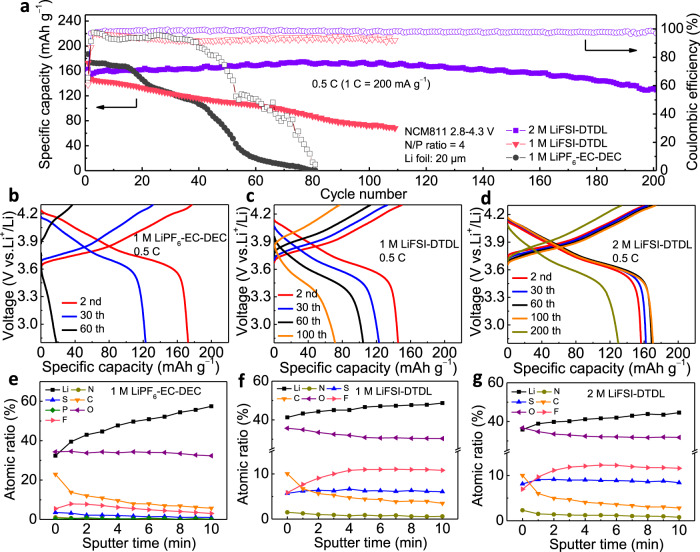
Electrochemical performance and SEI compositions of Li|NCM811 full cells. **a** Cycling performance of Li|NCM811 full cells at 0.5 C after the first formation cycle at 0.1 C in each electrolyte. Charge/discharge profiles of Li|NCM811 full cells at different cycles in 1 M LiPF_6_-EC-DEC (**b**), 1 M LiFSI-DTDL (**c**) and 2 M LiFSI-DTDL (**d**) electrolytes. Quantified atomic ratios of SEI at different sputter time in Li|NCM811 full cells after 30 cycles at 0.5 C with 1 M LiPF_6_-EC-DEC (**e**), 1 M LiFSI-DTDL (**f**) and 2 M LiFSI-DTDL (**g**) electrolytes.

Based on high voltage tolerance and excellent LMA performance of 1 M LiFSI-DTDL, Li metal full cells were assembled using high-nickel NCM811 cathode (5 mg cm^−2^) paired with thin 20 μm Li foil by employing negative/positive (N/P) capacity ratio of 4 with different electrolytes (Fig. 4a). Li|NCM811 full cell with 1 M LiFSI-DME could not be charged to the cutoff voltage of 4.3 V *vs*. Li/Li^+^ and exhibited serious voltage drop because of the constant DME decomposition (Supplementary Fig. 28). The full cell with traditional carbonate electrolyte of 1 M LiPF_6_ in ethylene carbonate and diethyl carbonate (v: v = 1: 1, 1 M LiPF_6_-EC-DEC) showed a massive capacity fading after 15 cycles at 0.5 C, mainly due to serious Li dendrite formation and electrolyte consumption. When the electrolyte was replaced with 1 M LiFSI-DTDL under the same conditions, cell performance improved markedly and kept a slower capacity decrease for 108 cycles, which is ascribed to the good compatibility of the electrolyte with the cathode and the stable SEI layer formation on LMA. Furthermore, the performance of Li|NCM811 full cell was improved considerably by increasing the salt concentration to 2 M. At this salt concentration, 84% of the original capacity was retained after 200 cycles at 0.5 C, as the higher salt concentration enables better SEI and cathode electrolyte interphase (CEI) on anode and cathode surfaces, respectively^16^. The charge/discharge profiles of Li|NCM811 full cells were shown in Fig. 4b–d. The voltage polarization increased drastically in the cell with 1 M LiPF_6_-EC-DEC after 30 cycles primarily due to unstable SEI and ‘dead’ Li formation along with constant electrolyte consumption. The voltage polarization was alleviated with 1 M LiFSI-DTDL through efficient LMA protection that suppresses parasitic reactions. Notably, with increased salt concentration, the voltage profiles of cell with 2 M LiFSI-DTDL did not show a significant degradation for 200 cycles owing to the concentration effect of salt, which enhances compatibility with both LMA and high voltage cathode. In addition, reproducibility test with three cells under the same conditions obtained similar cycling performance using 2 M LiFSI-DTDL electrolyte (Supplementary Fig. 29). Regarding the safety of the liquid electrolyte, flammability of the 1 M LiPF_6_-EC-DEC and 2 M LiFSI-DTDL electrolytes were compared (Supplementary Fig. 30). 2 M LiFSI-DTDL exhibited a weak flame owing to the presence of the fluorine atoms in the solvent structure as oppose to 1 M LiPF_6_-EC-DEC with a rather strong flame^33^. As for the practical applications, cells using high loading NCM811 cathode of 20 mg cm^−2^ paired with 20 μm Li foil (N/P ratio of 1) were further evaluated at 0.3 C with 2 M LiFSI-DTDL electrolyte (Supplementary Fig. 31). The rate performance of full cells with different electrolytes was also evaluated (Supplementary Fig. 32). Moreover, 3 M LiFSI-DTDL electrolyte was evaluated by Li|Cu half cell and Li|NCM811 full cell (Supplementary Fig. 33), which exhibited better stability than 2 M LiFSI-DTDL.

### Interface characterization

In order to understand the origin of the superior electrochemical performance of DTDL, comparative XPS analysis was conducted to systematically evaluate the SEI and CEI compositions in Li|NCM811 full cells after 30 cycles at 0.5 C. XPS concentration depth profiles of the SEI on LMA surface of Li|NCM811 full cells with different electrolytes are shown in Fig. 4e–g. In the 1 M LiPF_6_-EC-DEC electrolyte, SEI layer has much higher carbon content originating from organic components in the SEI. With increasing sputtering time, the amount of organic species decreased while the inorganic species increased (based on Li signal)^2^. This result proved that the SEI layer in the 1 M LiPF_6_-EC-DEC electrolyte is rich in organic species at its outer surface; the concentration of inorganic species increases with increasing depth below the surface. The enrichment of organic species at the outer surface is much less pronounced for the SEI layers formed in the 1 M and 2 M LiFSI-DTDL electrolytes. The concentration depth profiles of the SEI layers formed in the 1 M and 2 M LiFSI-DTDL electrolytes were found to be similar and exhibited a far more uniform SEI composition. The higher S and F atomic contents in the case of 2 M LiFSI-DTDL indicated more pronounced FSI anion participation in the SEI formation. Notably, the F 1*s* spectra of SEIs at different sputtering times exhibited a constant and high LiF content in DTDL electrolytes (Supplementary Fig. 34). The binding states and depth-dependent concentrations of the different Li, F, C, O, S species in the SEI layers (as resolved from the recorded spectra after each sputtering step by peak fitting) are given in the Supplementary Figs. 35–40. It follows that, the 1 M LiPF_6_-EC-DEC SEI layer is relatively inhomogeneous; the Li_2_O content increased while ROCO_2_Li decreased with increasing depth, which clearly showed non-uniform nature of SEI. On the contrary, every species, especially the inorganic ones (i.e., Li_2_O and LiF), have a roughly constant concentration for DTDL-based electrolyte. The Li_2_O content in 2 M LiFSI-DTDL reduced to about a half of that in 1 M LiFSI-DTDL, which is expected owing to the decreased free solvent molecules at high salt concentrations. The inorganic-rich and highly homogenous SEI in DTDL-based electrolytes alleviated continuous decomposition of electrolyte, which is beneficial for robust cycling. XPS spectra of CEI on NCM811 cathodes were shown in Supplementary Figs. 41 and 42. Compared to the C 1*s* spectrum of 1 M LiPF_6_-EC-DEC, one additional peak emerged at 293.3 eV when using 1 M and 2 M LiFSI-DTDL electrolytes, which is ascribed to the -CF_3_ functional group^34^. This result led us to conclude that DTDL participated in forming the CEI by its decomposition. The weaker CF_3_ peak formed in 2 M LiFSI-DTDL electrolyte indicated weakened decomposition of solvent compared to the 1 M LiFSI-DTDL. In the F 1*s* spectra, LiF-rich character of CEI was revealed in both 1 M and 2 M LiFSI-DTDL electrolytes, significantly differing from 1 M LiPF_6_-EC-DEC.

### Controlling the solvation structure

**Fig. 5:**
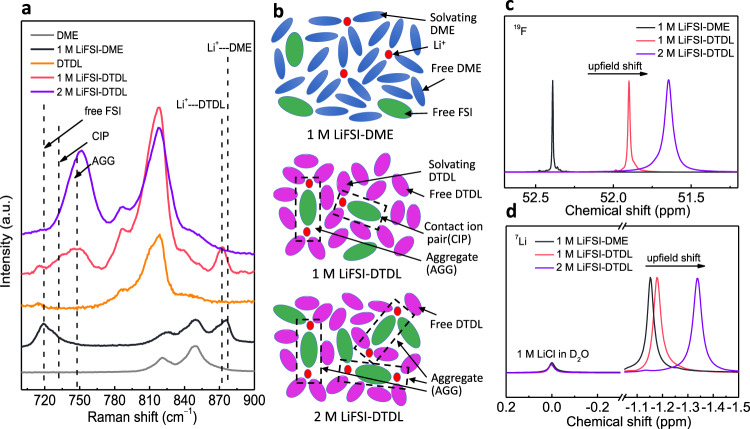
Electrolyte solvation structure analysis. **a** Raman spectra of solvents and electrolytes. **b** Schematic diagrams of Li^+^ coordination structures in different electrolytes. NMR spectra of ^19^F (**c**) and ^7^Li (**d**) of different electrolytes.

Excellent electrochemical performance of DTDL electrolyte can be explained by its electrolyte solvation structure (Fig. 5). Raman spectroscopy is an efficient tool to elucidate the solvation structure (Fig. 5a). Compared to the pure DME solvent, 1 M LiFSI-DME exhibited two additional peaks at 719 and 877 cm^−1^, assigned to the free FSI anion and Li^+^−DME coordination cluster, respectively. In the case of 1 M LiFSI-DTDL electrolyte, the free FSI anion peak weakened, but contact ion pair (CIP) and aggregate (AGG) peaks appeared^23^. The observation of the CIP and AGG in 1 M LiFSI-DTDL is well aligned with the formation of the FSI-derived inorganic SEI on the LMA surface. It should be mentioned that CIP and AGG usually exist in HCE and LHCE electrolytes to form stable SEI and CEI. Differently, this phenomenon can be realized at low salt concentrations by the tuned solvation ability of DTDL. Upon increasing the salt concentration, 2 M LiFSI-DTDL electrolyte, only the main peak of AGG at 750 cm^−1^ remained, which indicates a solvation structure rich in Li^+^-FSI^−^ clusters. Schematic diagrams in Fig. 5b depicts the solvation sheath in each electrolyte based on the results of Raman analysis. Compared to the DME-based electrolyte, the formation of CIP and AGG solvation structures in the DTDL based electrolytes decreased the LUMO energy of the anion, promoting FSI decomposition earlier than DTDL solvent, forming an anion-derived inorganic SEI^15^. Fourier-transform infrared (FTIR) spectra also further proved (Supplementary Fig. 43) the unique solvation structure in DTDL based electrolyte. Unlike DME, a strong FSI anion−Li^+^ coordination peak was detected in both 1 M and 2 M LiFSI-DTDL electrolytes. ^19^F and ^7^Li NMR spectra were recorded to evaluate ion bonding environment between FSI anion and Li^+^ in different electrolytes using 1 M LiCl-D_2_O as internal standard by coaxial NMR tube (Fig. 5c, d). The upfield shift of ^19^F and ^7^Li peak was observed going from 1 M LiFSI-DME to 1 M LiFSI-DTDL then to 2 M LiFSI-DTDL, an indication for enhanced FSI anion−Li^+^ coordination^11^, in a good agreement with the Raman results. Based on these results, it became evident that DTDL-based electrolyte showed more aggregate cluster to form FSI-derived inorganic-rich SEI layer and immobilize FSI anions through coordination to realize the high Li transference number, which explains their superior performance with LMAs.

## Discussion

In this work, a new class of fluorinated ether solvent was introduced. The rational molecular design enabled controlled solvation ability and enhanced tolerance against high voltages. Using a single-solvent of DTDL and a single-salt LiFSI to constitute an electrolyte, we achieved a high oxidation stability up to 5.5 V *vs*. Li/Li^+^ and high Li^+^ transference number of 0.75. Based on the unique solvation sheath, strong aggregate cluster of FSI anion-coordinated Li^+^ was formed at low concentrations, leading to a highly robust cycling performance in both Li|Cu half-cell and Li|NCM 811 full cell configurations. The molecular design of DTDL that manipulates solvation structure points to the promising nature of fluorinated ether for upcoming LMBs.

## Methods

### Materials

Trichloroacetonitrile was purchased from Apollo Scientific and distilled before using. Anhydrous DME, sodium borohydride (NaBH_4_), sodium methoxide and 1 M LiPF_6_-EC-DEC were purchased from Sigma-Aldrich. Sulfuric acid (95%), toluene and methanol were purchased from Fisher Scientific. Methyl trifluoropyruvate was purchased from Fluorochem. p-Toluenesulfonic acid was purchased from TCI and dried by using Dean-Stark apparatus in toluene. Anhydrous THF was taken from a solvent drying system. Battery grade LiFSI, polyvinylidene fluoride (PVDF), Super-P, polypropylene separator (2400), NCM 811 powder and laminate (20 mg cm^−1^) were purchased from Guangdong Canrd New Energy Technology Co., Ltd. Ultra-thin Li foil (20 μm) was purchased from China Energy Lithium Co., Ltd.

### Syntheses

#### TMOC

TMOC was synthesized by using a modified literature procedure^24^. To a two-neck round bottomed flask, 350 mL of methanol was added, followed by the slow addition of sodium methoxide (108 g, 2 mol). Freshly distilled trichloroacetonitrile (72 g, 0.5 mol) was added to this solution by using a syringe pump under Ar atmosphere and the resulting mixture was refluxed for 3 h. Afterwards, about 60% of the solvent was removed by fractional distillation. After cooling the solution, 600 mL of an aqueous 3 wt% hydrogen peroxide solution was slowly added at room temperature. The mixture was stirred for 1 h and 200 mL cyclohexane was added. After extraction, the organic phase was subjected to a fractional distillation using a 50 cm fraction column, and the product was collected at 112–114 °C. Yield: 30–40%. ^1^H NMR (300 MHz, CDCl_3_) δ_H_ = 3.27 (s, 12H) ppm; ^13^C NMR (75 MHz, CDCl_3_) δ_C_ = 120.7 (s), 50.2 (s) ppm.

#### TFPD

The synthetic procedure was optimized based on a previously reported patent^25^. In total, 50 g (0.32 mol) methyl trifluoropyruvate was added using a syringe pump at the rate of 20 mL h^−1^ to the solution of 11.8 g (0.32 mol) NaBH_4_ in 50 mL anhydrous THF under Ar atmosphere while maintaining the temperature at about 50 °C. Following the addition, the mixture was refluxed for 1 h. After cooling, 12 mL of H_2_O was slowly added and the mixture was further refluxed for 1 h. The reaction mixture was slowly acidified using 95% sulfuric acid, and 50 mL H_2_O was added at room temperature, followed by another reflux for 1 h. Then, the solution was extracted using ethyl acetate and the organic phase was washed with water three times. Organic phase was subjected to vacuum distillation. The product was collected at 41 °C and 1.2 Torr. Yield: 44–68%. ^1^H NMR (400 MHz, CD_3_OD) δ_H_ = 3.96 (m, 1H), 3.73 (m, 1H), 3.63 (m, 1H) ppm; ^19^F NMR (376 MHz, CD_3_OD) δ_F_ = –79.29 (d, J = 6.81 Hz, 3F) ppm; ^13^C NMR (100 MHz, CD_3_OD) δ_C_ = 126.4 (q, J = 281.70 Hz), 72.1 (q, J = 29.34 Hz), 61.8 (s) ppm.

#### DTDL

To a two-neck round bottomed flask equipped with a Dean-Stark apparatus, *p*-toluenesulfonic acid (0.243 g, 1.3 mmol) and TMOC (15 g, 0.11 mol) were added and the reaction mixture was heated to 110 °C. TFPD (13 g, 0.1 mol) was slowly (1 mL h^−1^) added to this mixture using a syringe pump. The mixture was stirred for 3 h at 110 °C. Afterwards, the product was collected by vacuum distillation at 53–54 ^o^C and 12 Torr. Yield: 80%. ^1^H NMR (400 MHz, DMSO) δ_H_ = 4.97 (m, 1H), 4.32 (m, 1H), 4.19 (m, 1H), 3.33 (s, 3H), 3.31 (s, 3H) ppm; ^19^F NMR (376 MHz, DMSO) δ_F_ = –77.26 (d, J = 6.81 Hz, 3F) ppm; ^13^C NMR (100 MHz, DMSO) δ_C_ = 128.5 (s), 123.8 (q, J = 280.23 Hz), 71.6 (q, J = 33.0 Hz), 63.4 (s), 51.5 (s), 51.3 (s) ppm. Electron ionization-mass spectrometry (EI-MS) calculated [M+H]^+^: 203.0526; found: 203.0525.

#### Electrolytes

All electrolytes were prepared in a glove box. For 1 M LiFSI-DME and 1 M LiFSI-DTDL, LiFSI (187 mg) was dissolved in 1 mL DME or DTDL. For 2 M LiFSI-DTDL, LiFSI (374 mg) was dissolved in 1 mL DTDL. All electrolytes were stirred for 4 hours at room temperature.

### Material characterizations

^1^H, ^19^F and ^13^C NMR spectra were recorded on 300 or 400 MHz Bruker NMR spectrometer at ambient temperature. ^19^F and ^7^Li NMR spectra of electrolytes were recorded on 500 MHz Bruker NMR spectrometer at ambient temperature using 1 M LiCl-D_2_O as an internal standard in a coaxial NMR tube. Viscosity of the solvents and electrolytes were measured by PHYSICA MCR 300 at 25 °C. Raman spectroscopy analyses were conducted on Renishaw inVia confocal Raman microscope using 785 nm laser. Fourier transform infrared (FTIR) spectra were obtained by Bruker TENSOR II. Morphology of plated Li was imaged on field emission scanning electron microscopy (FE-SEM, Tescan Mira3 LM FE). Li metal was plated at 0.5 mA cm^−2^ with a cutoff capacity of 0.5 mAh cm^−2^ on 3 mm diameter amorphous carbon film-coated Cu grids using 1 M and 2 M LiFSI-DTDL electrolytes. All the samples were protected under inert atmosphere during transfer and load. TEM analyses were performed on a TEM ThermoFisher Talos F200S equipped with a Ceta camera and operated at 200 kV. Bright-field images were recorded using a low dose electron beam at a rate of 9 e Å^−2^ s^−1^ for ~15 s. X-ray photoelectron spectroscopy (XPS) measurements of SEI and CEI were carried out with a VG ESCALAB 220iXL spectrometer (Thermo Fisher Scientific) using focused monochromatized Al Kα radiation (1486.6 eV) with a beam size of ~500 μm^2^ (power of 150 W). The spectrometer was calibrated on the clean silver surface by measuring the Ag 3*d*_5/2_ peak at a binding energy (BE) of 368.25 eV with a full width at half maximum (FWHM) of 0.78 eV. The calibration of the binding energy peak positions was applied on the C 1*s* located at 284.8 eV. For the depth profiles on cycled Li, XPS analysis was performed using a PHI Quantes spectrometer (ULVAC-PHI), as equipped with a conventional low-energy Al-Kα source (1486.6 eV) and a high energy Cr-Kα (5414.7 eV) X-ray source. Composition depth profiles were recorded by employing alternating cycles of XPS analysis (Al-Kα at 51 W; beam diameter ~200 μm) and sputtering with a focused 1 keV Ar beam, rastering an area of 2x2 mm^2^. During each measurement cycle, the Li 1*s*, P 2*p*, S 2*p*, C 1*s*, N 1*s*, O 1*s* and F 1*s* regions were recorded with a step size of 0.13 eV and a pass energy of 69 eV. The etch rate was calibrated to be 2.3 nm min^−1^ on a 100 nm Ta_2_O_5_/Ta reference sample. Chemical state analysis and quantification of each element (i.e. Li, P, S, C, N, O and F) were performed by constrained peak fitting of the corresponding Shirley background corrected spectra with one or more symmetrical, mixed Gaussian–Lorentzian line shape functions, using the MultiPak 9.9 software of Ulvac-Phi. The binding energy was calibrated by C 1*s* peak of adventitious carbon of 284.6 eV. All electrode samples were rinsed with anhydrous DME and transferred by argon protection. HOMO-LUMO energy and ESP were calculated using density functional theory conducted at B3LYP/6-311++G** by Spartan 14.

### Electrochemical measurements

All cells were assembled in a glove box using Swagelok-cell or 2032-type coin-cell with an electrolyte amount of 40 μL except high loading full cells with 60 μL using Celgard 2400 as the separator. Stainless steel (SS) is widely used as a counter electrode to detect the oxidation stability of conventional electrolytes, but it exhibits serious corrosion in the presence of LiFSI salt at high voltages. Accordingly, Al counter electrode was used to assess the oxidation stability of electrolytes by avoiding corrosion. CV was conducted by using Li|SS and Li|Al half-cells at a scan rate of 1 mV s^–1^. LSV analysis was conducted by using Li|Al half cell from OCV to 6 V at a scan rate of 5 mV s^–1^. EIS was performed by using Li|Cu half-cell with a disturbance amplitude of 5 mV in the frequency range of 100 kHz to 10 mHz. Ionic conductivities of electrolytes were measured via EIS by SS|SS blocking cell from –20 °C to 60 °C with an amplitude of 5 mV in the frequency range of 500 kHz to 100 mHz. Lithium ion transference number (${{{{{{\rm{t}}}}}}}_{{{{{{\rm{Li}}}}}}}^{+}$) was measured on the Li|Li symmetric cell^27^. The initial and steady polarization currents (*I*^*o*^ and *I*^*ss*^) were measured under a polarization potential of 10 mV. The initial and steady bulk resistances (*R*_*b*_^*o*^ and *R*_*b*_^*ss*^) and interfacial resistances (*R*_*i*_^*o*^ and *R*_*i*_^*ss*^) were obtained by EIS before and after the above potentiostatic polarization. The (${{{{{{\rm{t}}}}}}}_{{{{{{\rm{Li}}}}}}}^{+}$) was calculated by the following equation:

1 $${{{{{{\rm{t}}}}}}}_{{{{{{\rm{Li}}}}}}}^{+}=\frac{{I}^{{ss}}{R}_{b}^{{ss}}(\triangle V-{I}^{o}{R}_{i}^{o})}{{I}^{o}{R}_{b}^{o}(\triangle V-{I}^{{ss}}{R}_{i}^{{ss}})}$$

Above tests were carried on a VMP3 (Bio Logic Science Instruments) electrochemical working station. CE cycling measurements were conducted in Li|Cu half cells, which were cycled at 0.05 mA in the potential range of 0-1.5 V for 10 cycles to stabilize the interface. Then Li was plated at a constant capacity on Cu foil and then stripped at a constant current density until the potential reached to 1 V. Galvanostatic cycling performances of Li|Li symmetric cells were measured in Li|Cu half cells with pre-plated 10 mAh cm^–2^ Li on Cu foil at 0.5 mA cm^–2^ current density, then the cells were cycled at 0.5 mA cm^–2^ of 1 mAh cm^–2^ capacity for each charge and discharge. For Li|NCM811 full cell, Al coated cathode case and a piece Al foil were used to suppress electrolyte corrosion on stainless-steel. NCM811 electrode was prepared by mixing the NCM811 active material, Super-P and PVDF at the weight ratio of 8: 1: 1 in an anhydrous *N*-methyl-2-pyrrolidone (NMP), being cast on the carbon-coated Al foil, with the active loading of 5 mg cm^–2^. After pairing with 20 μm Li foil (N/P ratio of 4), the cells were cycled at 0.5 C between 2.8-4.3 V. High loading NCM811 (20 mg cm^–2^) paired with 20 μm Li foil (N/P ratio of 1) was tested at 0.3 C between 2.8-4.3 V, with a CV step at 4.3 V till the current dropped to 0.05 C (1 C = 200 mA g^–1^). All cycling tests were performed on a LAND 2001 A battery system.

## Supplementary information

Supplementary Information

## Data Availability

The data generated in this study have been deposited in the Zenodo database under accession code 6113559.

## References

[1] Yu Z (2020). Molecular design for electrolyte solvents enabling energy-dense and long-cycling lithium metal batteries. Nat. Energy.

[2] Cao X (2019). Monolithic solid–electrolyte interphases formed in fluorinated orthoformate-based electrolytes minimize Li depletion and pulverization. Nat. Energy.

[3] Zhang JG, Xu W, Xiao J, Cao X, Liu J (2020). Lithium Metal Anodes with Nonaqueous Electrolytes. Chem. Rev..

[4] Lee Y-G (2020). High-energy long-cycling all-solid-state lithium metal batteries enabled by silver–carbon composite anodes. Nat. Energy.

[5] Lin D, Liu Y, Cui Y (2017). Reviving the lithium metal anode for high-energy batteries. Nat. Nanotechnol..

[6] Niu C (2019). High-energy lithium metal pouch cells with limited anode swelling and long stable cycles. Nat. Energy.

[7] Choi JW, Aurbach D (2016). Promise and reality of post-lithium-ion batteries with high energy densities. Nat. Rev. Mater..

[8] Liu J (2019). Pathways for practical high-energy long-cycling lithium metal batteries. Nat. Energy.

[9] Fang C, Wang X, Meng YS (2019). Key issues hindering a practical lithium-metal anode. Trends Chem..

[10] Zheng X (2021). Critical effects of electrolyte recipes for Li and Na metal batteries. Chem.

[11] Amanchukwu CV (2020). A new class of ionically conducting fluorinated ether electrolytes with high electrochemical stability. J. Am. Chem. Soc..

[12] Yamada Y, Wang J, Ko S, Watanabe E, Yamada A (2019). Advances and issues in developing salt-concentrated battery electrolytes. Nat. Energy.

[13] Fang C (2019). Quantifying inactive lithium in lithium metal batteries. Nature.

[14] Cao X (2021). Effects of fluorinated solvents on electrolyte solvation structures and electrode/electrolyte interphases for lithium metal batteries. Proc. Natl. Acad. Sci. USA.

[15] Ren X (2020). Role of inner solvation sheath within salt-solvent complexes in tailoring electrode/electrolyte interphases for lithium metal batteries. Proc. Natl. Acad. Sci. USA.

[16] Qian J (2015). High rate and stable cycling of lithium metal anode. Nat. Commun..

[17] Chen S (2018). High-voltage lithium-metal batteries enabled by localized high-concentration electrolytes. Adv. Mater..

[18] Ren X (2018). Localized high-concentration sulfone electrolytes for high-efficiency lithium-metal batteries. Chem.

[19] Yao YX (2021). Regulating interfacial chemistry in lithium‐ion batteries by a weakly solvating electrolyte. Angew. Chem..

[20] Zeng Z (2018). Non-flammable electrolytes with high salt-to-solvent ratios for Li-ion and Li-metal batteries. Nat. Energy.

[21] Wang J (2016). Superconcentrated electrolytes for a high-voltage lithium-ion battery. Nat. Commun..

[22] Holoubek J (2021). Tailoring electrolyte solvation for Li metal batteries cycled at ultra-low temperature. Nat. Energy.

[23] Yamada Y, Yaegashi M, Abe T, Yamada A (2013). A superconcentrated ether electrolyte for fast-charging Li-ion batteries. Chem. Commun..

[24] Kantlehner W (1977). Die präparative Chemie der O- und N-funktionellen Orthokohlensäure-Derivate. Synthesis.

[25] Koh, M., Yamauchi, A., Tanaka, M. & Saita, T. Preparation of fluorine-containing diols by hydride reduction of polyfluorooxocarboxylate esters. JP patent 2008230970A (2008).

[26] Endo T, Okawara M (1984). A facile synthesis of spiro orthocarbonates. Synthesis.

[27] Abraham K, Jiang Z, Carroll B (1997). Highly conductive PEO-like polymer electrolytes. Chem. Mater..

[28] Sand HJ (1901). On the concentration at the electrodes in a solution, with special reference to the liberation of hydrogen by electrolysis of a mixture of copper sulphate and sulphuric acid. Lond. Edinb. Dublin Philos. Mag. J. Sci..

[29] Jeon DH (2019). Wettability in electrodes and its impact on the performance of lithium-ion batteries. Energy Storage Mater..

[30] Jiao S (2018). Behavior of lithium metal anodes under various capacity utilization and high current density in lithium metal batteries. Joule.

[31] Miao R (2016). A new ether-based electrolyte for dendrite-free lithium-metal based rechargeable batteries. Sci. Rep..

[32] Yoo DJ, Yang S, Kim KJ, Choi JW (2020). Fluorinated aromatic diluent for high‐performance lithium metal batteries. Angew. Chem. Int Ed..

[33] Fan X (2018). Non-flammable electrolyte enables Li-metal batteries with aggressive cathode chemistries. Nat. Nanotechnol..

[34] Tan S (2012). Tris (hexafluoro-iso-propyl) phosphate as an SEI-forming additive on improving the electrochemical performance of the Li [Li0. 2Mn0. 56Ni0. 16Co0. 08] O2 cathode material. J. Electrochem. Soc..

