# Supplementary material for: Fluorinated ether electrolyte with controlled solvation structure for high voltage lithium metal batteries
Source: Nat Commun. 2022 May 6;13:2575. doi: 10.1038/s41467-022-29199-3 (PMC9076822; doi:10.1038/s41467-022-29199-3)
Supplement: Supplementary file 1 — Supplementary Information [file 41467_2022_29199_MOESM1_ESM.pdf]

Supplementary Information for

Fluorinated Ether Electrolyte with Controlled Solvation Structure for High Voltage Lithium Metal Batteries

Yan Zhao[1†], Tianhong Zhou[1†], Timur Ashirov[1], Mario El Kazzi[2], Claudia Cancellieri[3], Lars P. H. Jeurgens[3], Jang Wook Choi[4]*, Ali Coskun[1]*

[1]Department of Chemistry, University of Fribourg, Chemin de Musee 9, Fribourg 1700, Switzerland.

[2]Electrochemistry Laboratory, Paul Scherrer Institut, Villigen 5232, Switzerland.

[3]Laboratory for Joining Technologies and Corrosion, Swiss Federal Laboratories for Materials Science and Technology, Empa, Überlandstrasse 129, Dübendorf CH 8600, Switzerland.

[4]School of Chemical and Biological Engineering, Department of materials science and engineering, and Institute of Chemical Processes, Seoul National University, 1 Gwanak-ro, Gwanak-gu, Seoul 08826, Republic of Korea.

*Correspondence to:

Prof. Ali Coskun, E-mail: ali.coskun@unifr.ch

Prof. Jang Wook Choi, E-mail: jangwookchoi@snu.ac.kr

[†]These authors contributed equally to this work.

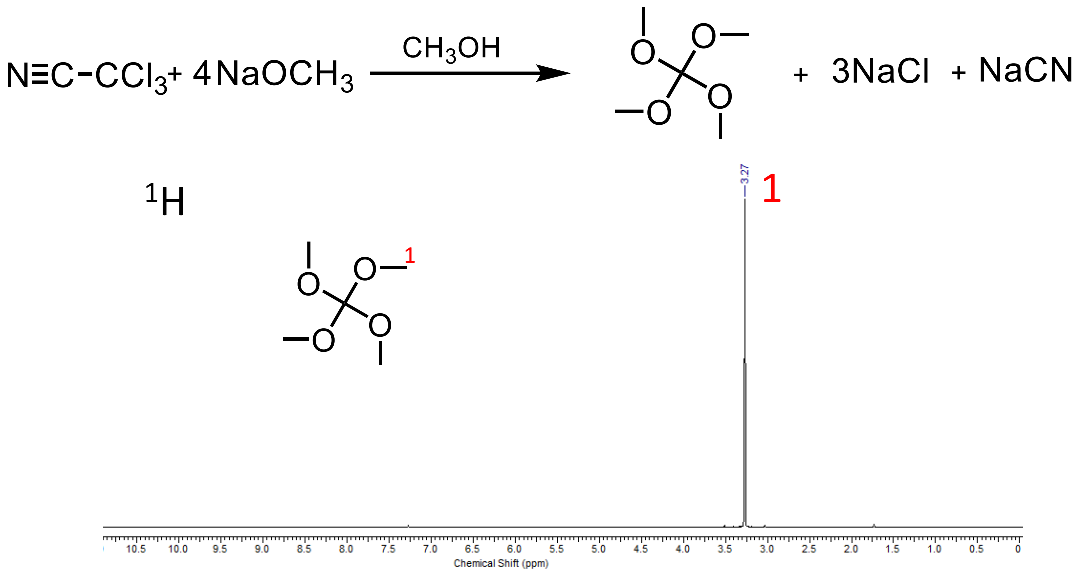

Supplementary Figure 1. Synthetic scheme for the preparation of tetramethylorthocarbonate (TMOC) and its ^{1}H NMR (300 MHz, $CDCl_3$, 298K) spectrum.

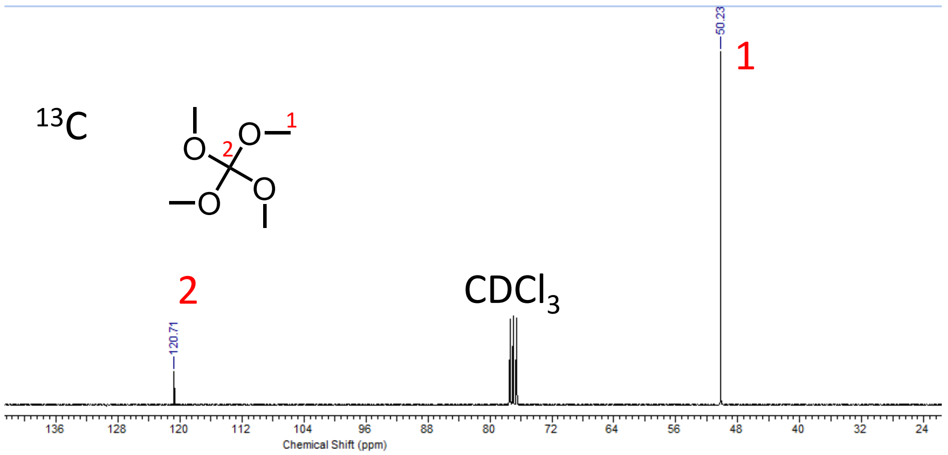

Supplementary Figure 2. ^{13}C NMR (75 MHz, $CDCl_3$, 298K) spectrum of TMOC.

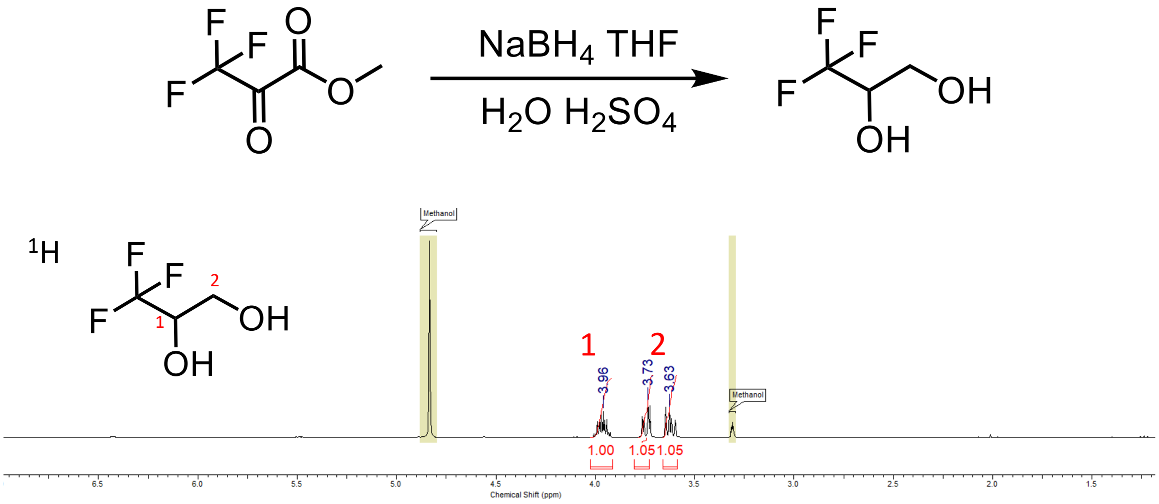

Supplementary Figure 3. Synthetic scheme for the preparation of 1,1,1-trifluoro-2,3-propanediol (TFPD) and its ^{1}H NMR (400 MHz, CD_3OD, 298K) spectrum.

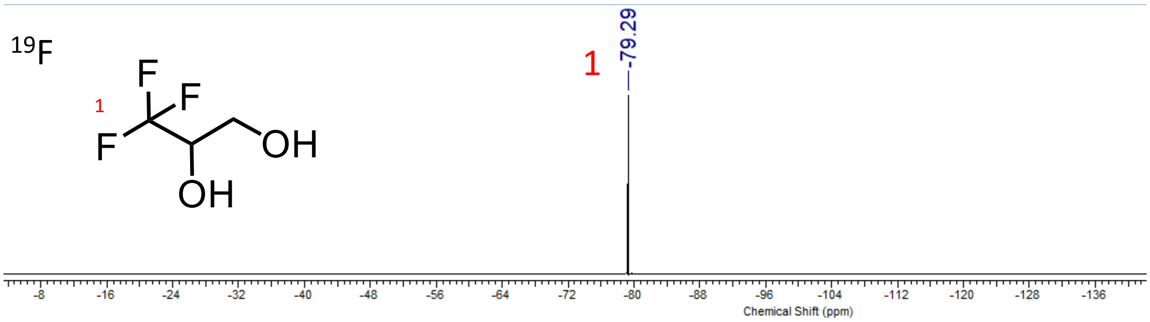

Supplementary Figure 4. ^{19}F NMR (376 MHz, CD_3OD, 298K) spectrum of TFPD.

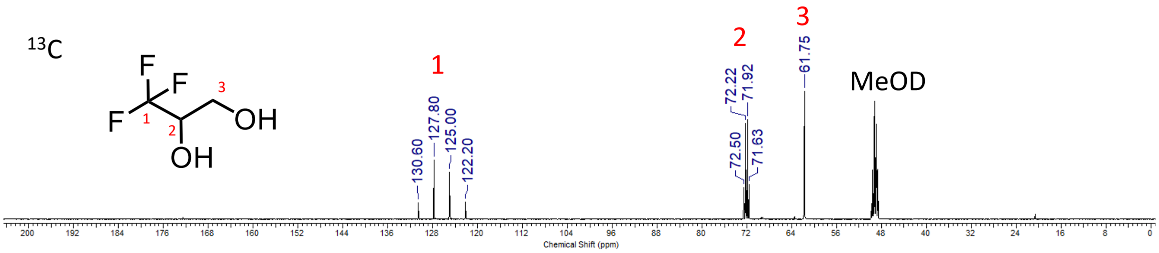

Supplementary Figure 5. ^{13}C NMR (100 MHz, CD_3OD, 298K) spectrum of TFPD.

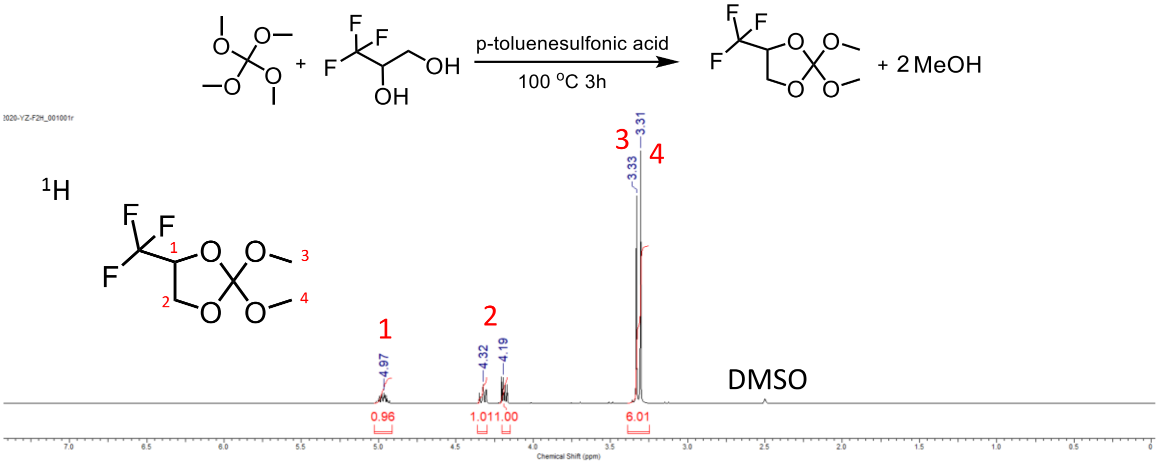

Supplementary Figure 6. Synthetic scheme for the preparation of DTDL and its ^{1}H NMR (400 MHz, DMSO-d_6, 298K) spectrum.

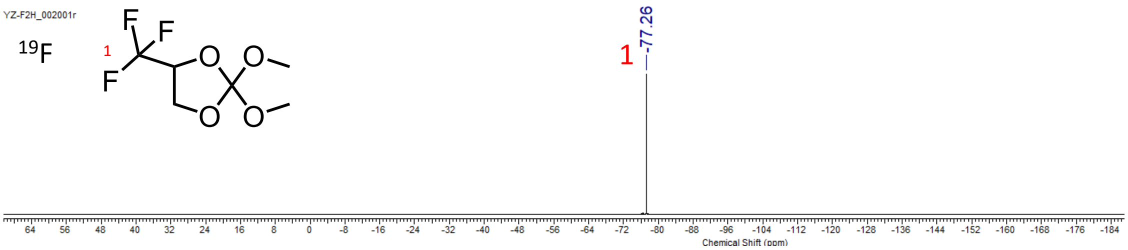

Supplementary Figure 7. ^{19}F NMR (376 MHz, DMSO-d_6, 298K) spectrum of DTDL.

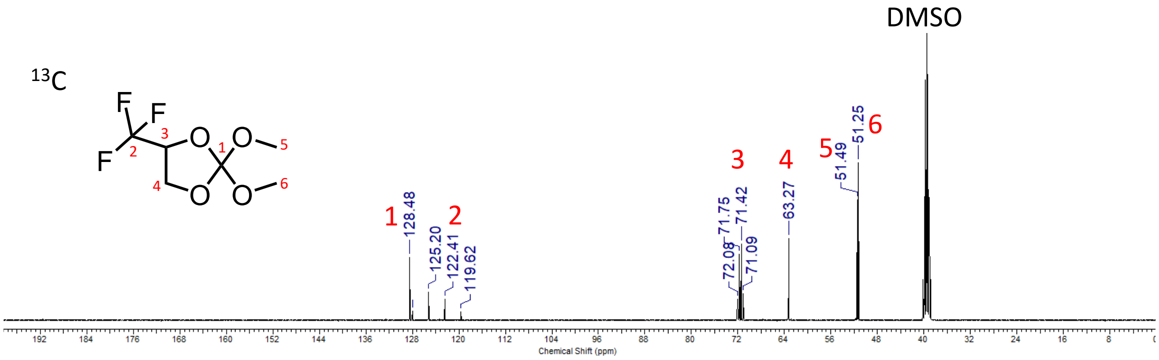

Supplementary Figure 8. ^{13}C NMR (100 MHz, DMSO-d_6, 298K) spectrum of DTDL.

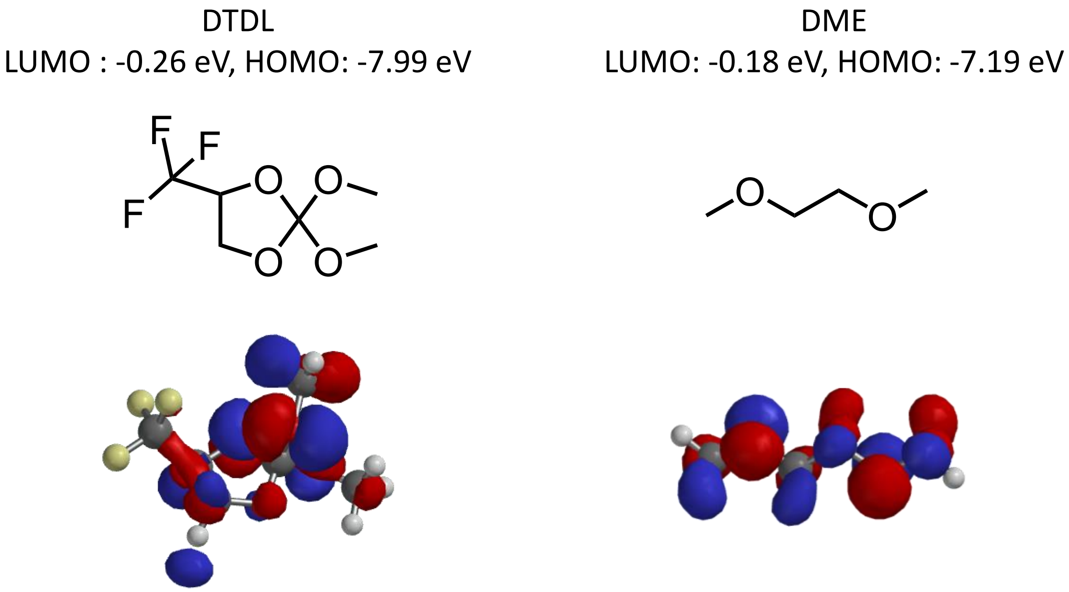

Supplementary Figure 9. HOMO-LUMO energy level calculations of DTDL and DME using DFT conducted at B3LYP/ 6-311++G**.

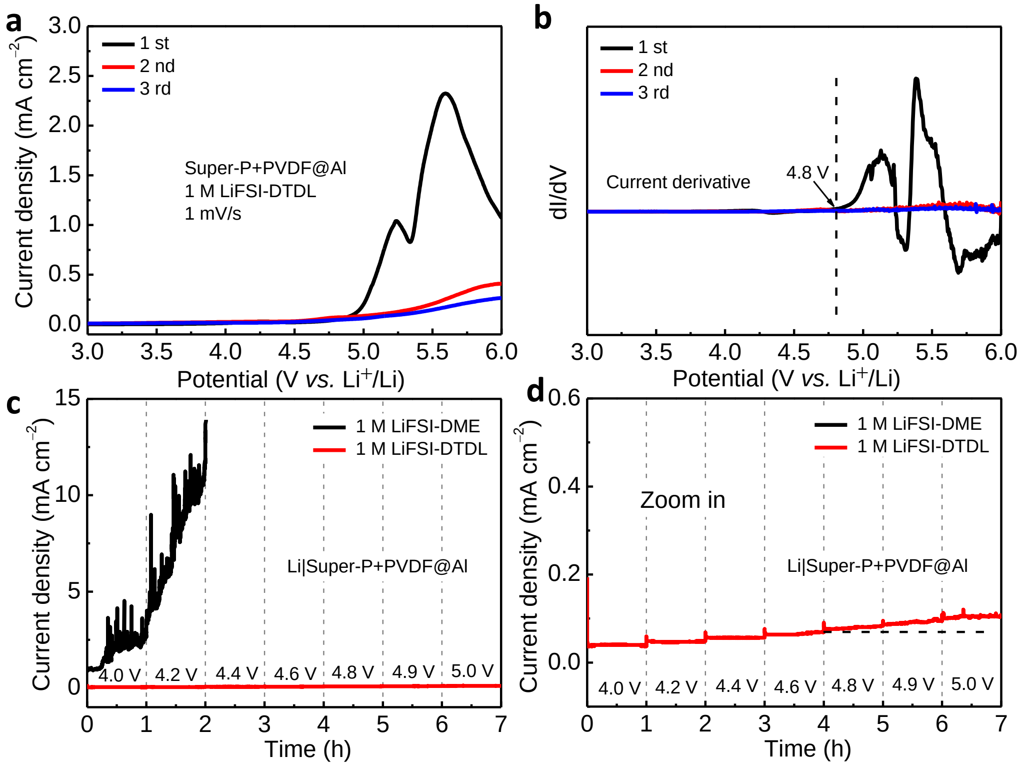

Supplementary Figure 10. Oxidation stability of 1 M LiFSI-DTDL in Li|Super-P+PVDF@Al cell tested by linear sweep voltammetry (LSV) over three cycles (a) and its current derivative (b), the scan rate is 1 mV s^{-1}; Potentiostatic polarization test of Li|Super-P+PVDF@Al cells with different electrolytes (c) and zoom-in (d). The weight ratio of Super-P and PVDF is 1:1.

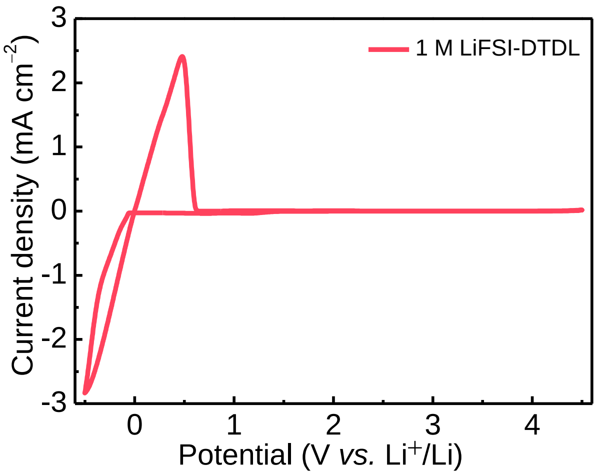

Supplementary Figure 11. CV curve of Li|SS half-cell in 1 M LiFSI-DTDL electrolyte at a scan rate of 1 mV s^{-1}.

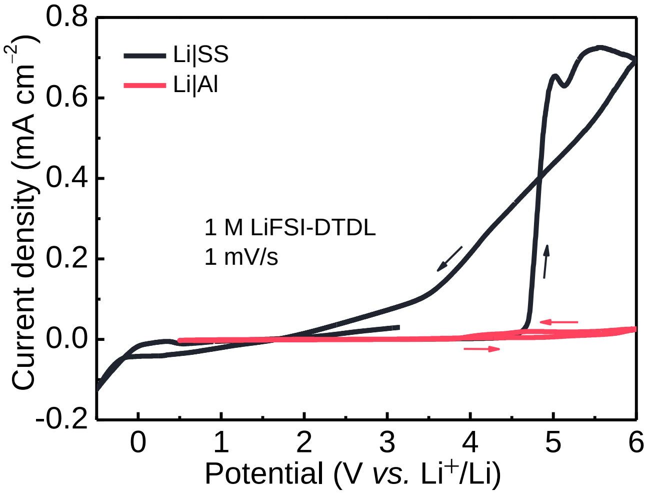

Supplementary Figure 12. Comparison of CV curve in Li|SS and Li|Al half-cell in 1 M LiFSI-DTDL electrolyte at a scan rate of 1 mV s^{-1}.

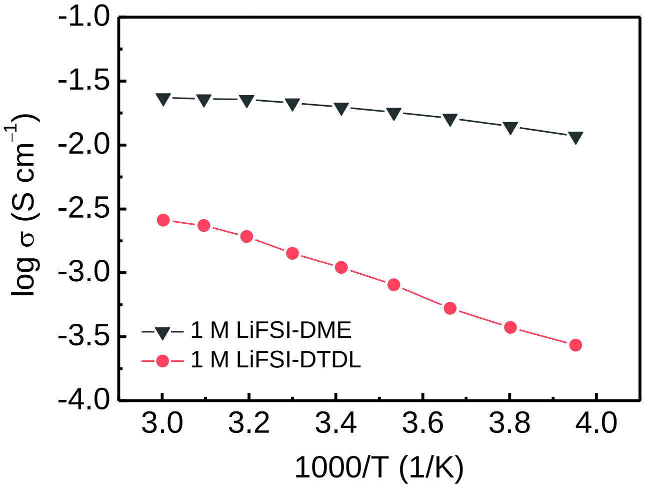

Supplementary Figure 13. Ionic conductivity of 1 M LiFSI-DME and 1 M LiFSI-DTDL electrolytes at different temperatures.

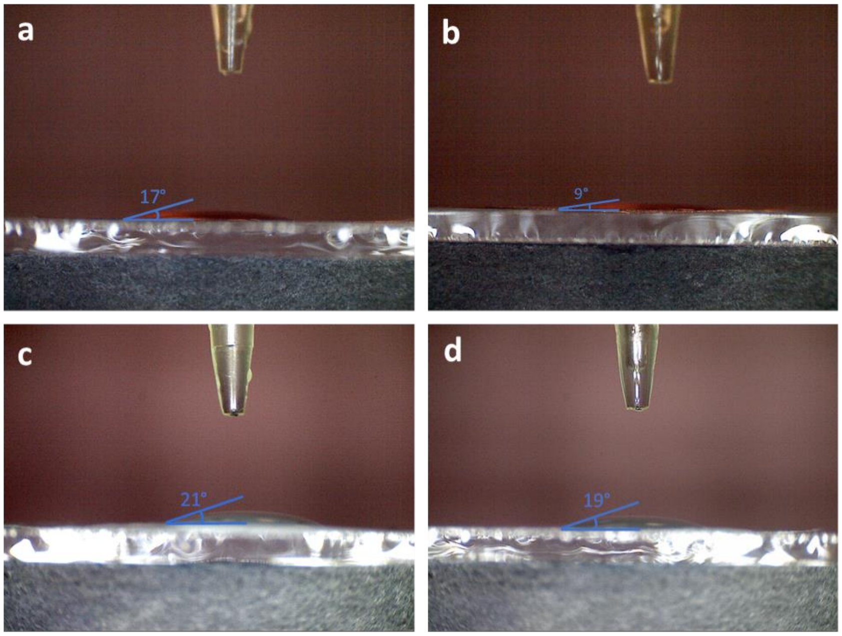

Supplementary Figure 14. Contact angle measurements on Cu foil for 1 M LiFSI-DME (a) and 1 M LiFSI-DTDL (b) electrolytes and on separator for 1 M LiFSI-DME (c) and 1 M LiFSI-DTDL (d) electrolytes.

Supplementary Table 1. The property comparisons of solvents and electrolytes.

Category	DME	1 M LiFSI-DME	DTDL	1 M LiFSI-DTDL
Viscosity (cP, 25 °C)	0.4	1.2	2.4	8
Boiling point (°C)	85	–	53-54 (12 Torr)	–
Density (g mL^{-1})	0.87	1.07	1.31	1.38
Ionic conductivity (mS cm^{-1}, 30 °C)	–	21.3	–	1.4
HOMO (eV)	-7.19	–	-7.99	
LUMO (eV)	-0.18	–	-0.26	–
Li^+ transference number	–	0.39	–	0.75
Oxidation stability (V) (Li|Al)	–	4	–	5.5

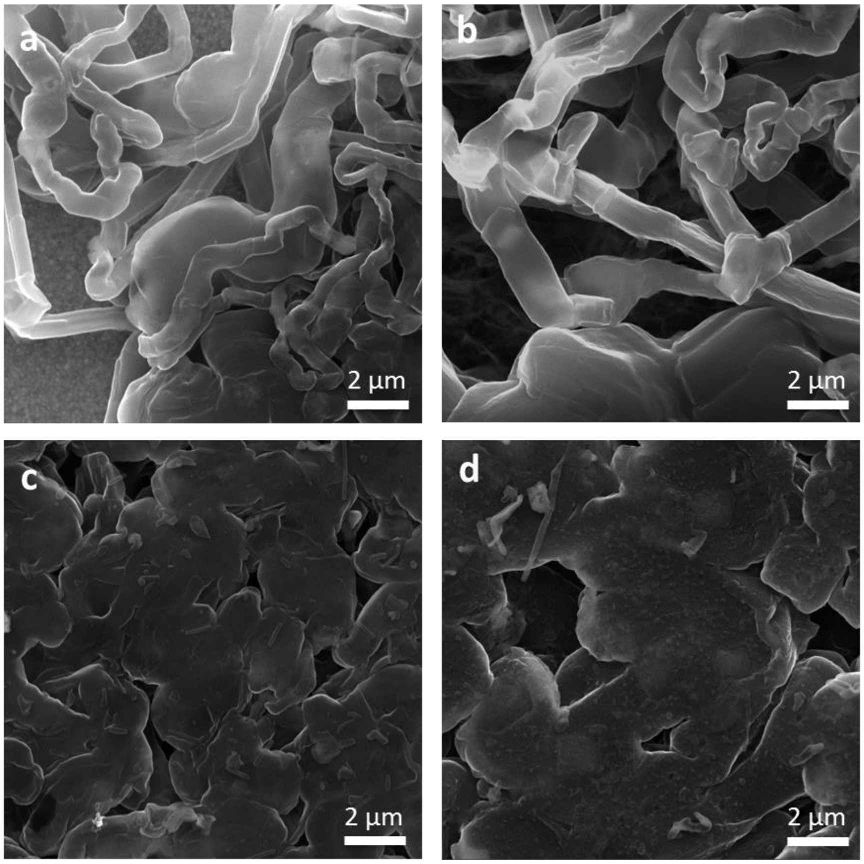

Supplementary Figure 15. a, c, High resolution SEM images of the Li plating morphology after the first cycle in 1 M LiFSI-DME (a) and 1 M LiFSI-DTDL (c) electrolytes. b, d, SEM images of the plated Li after 20 cycles in 1 M LiFSI-DME (b) and 1 M LiFSI-DTDL (d). All Li|Cu half cells were cycled at 1 mA cm^{-2} with a cutoff capacity of 1 mAh cm^{-2}.

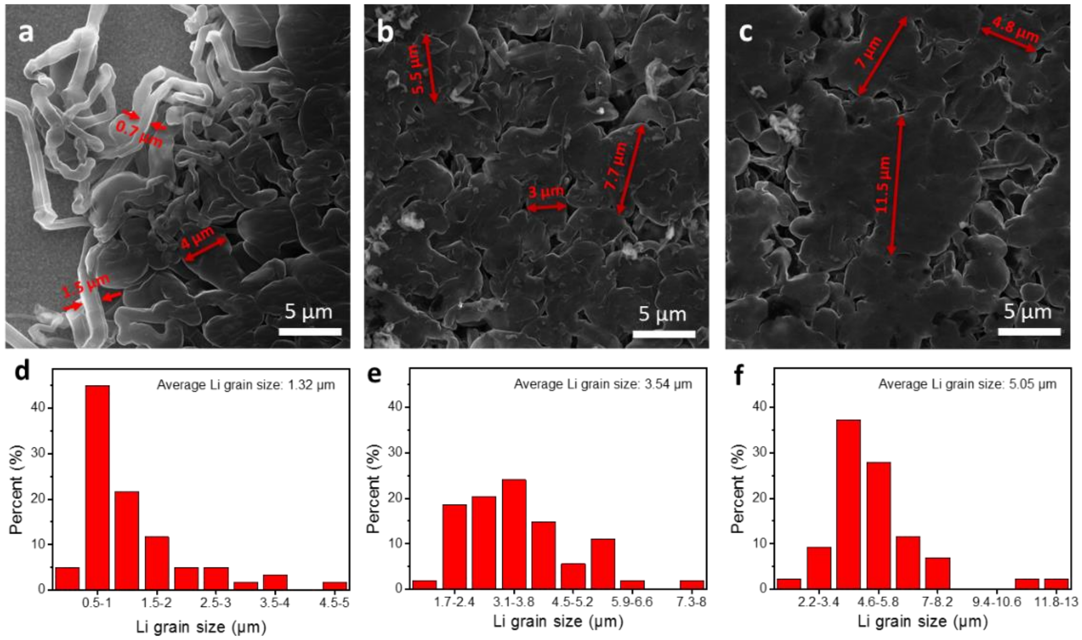

Supplementary Figure 16. SEM images and the corresponding Li grain size distributions after the first plating in 1 M LiFSI-DME (a, d), 1 M LiFSI-DTDL (b, e) and 2 M LiFSI-DTDL (c, f) electrolytes at 1 mA cm^{-2} with a cutoff capacity of 1 mAh cm^{-2}.

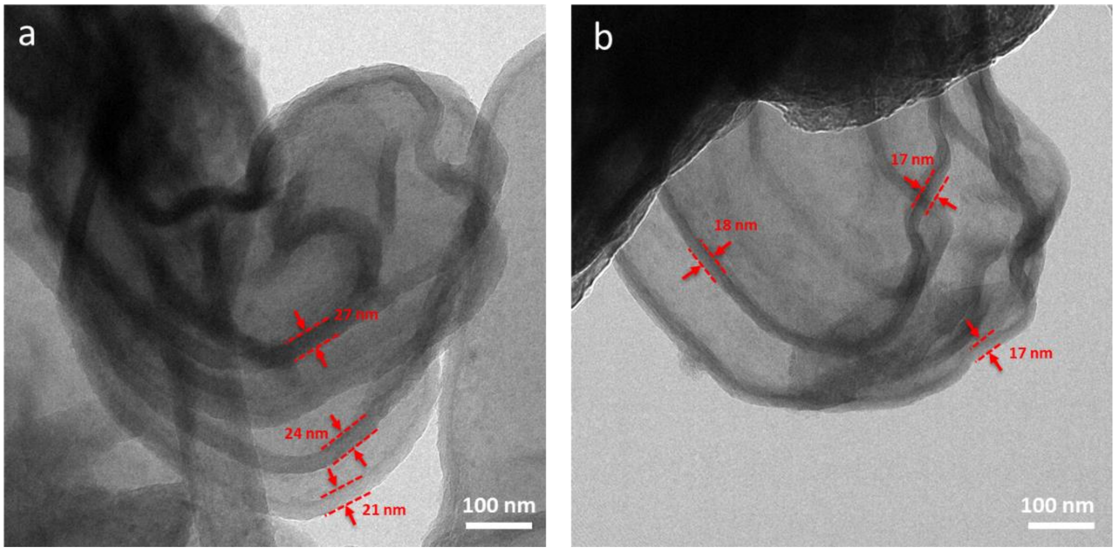

Supplementary Figure 17. Bright-field TEM images of plated Li on Cu grid in 1 M LiFSI-DTDL (a) and 2 M LiFSI-DTDL (b) electrolytes at 0.5 mA cm^{-2} with a cutoff capacity of 0.5 mAh cm^{-2}.

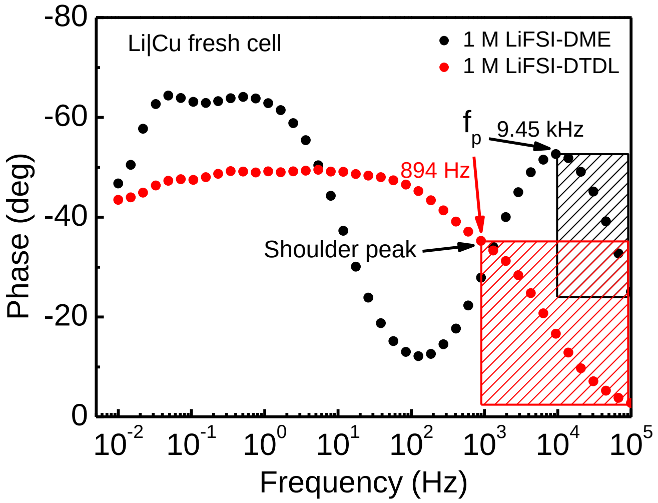

Supplementary Figure 18. The Bode plots of Li|Cu fresh cells with 1 M LiFSI-DME and 1 M LiFSI-DTDL.

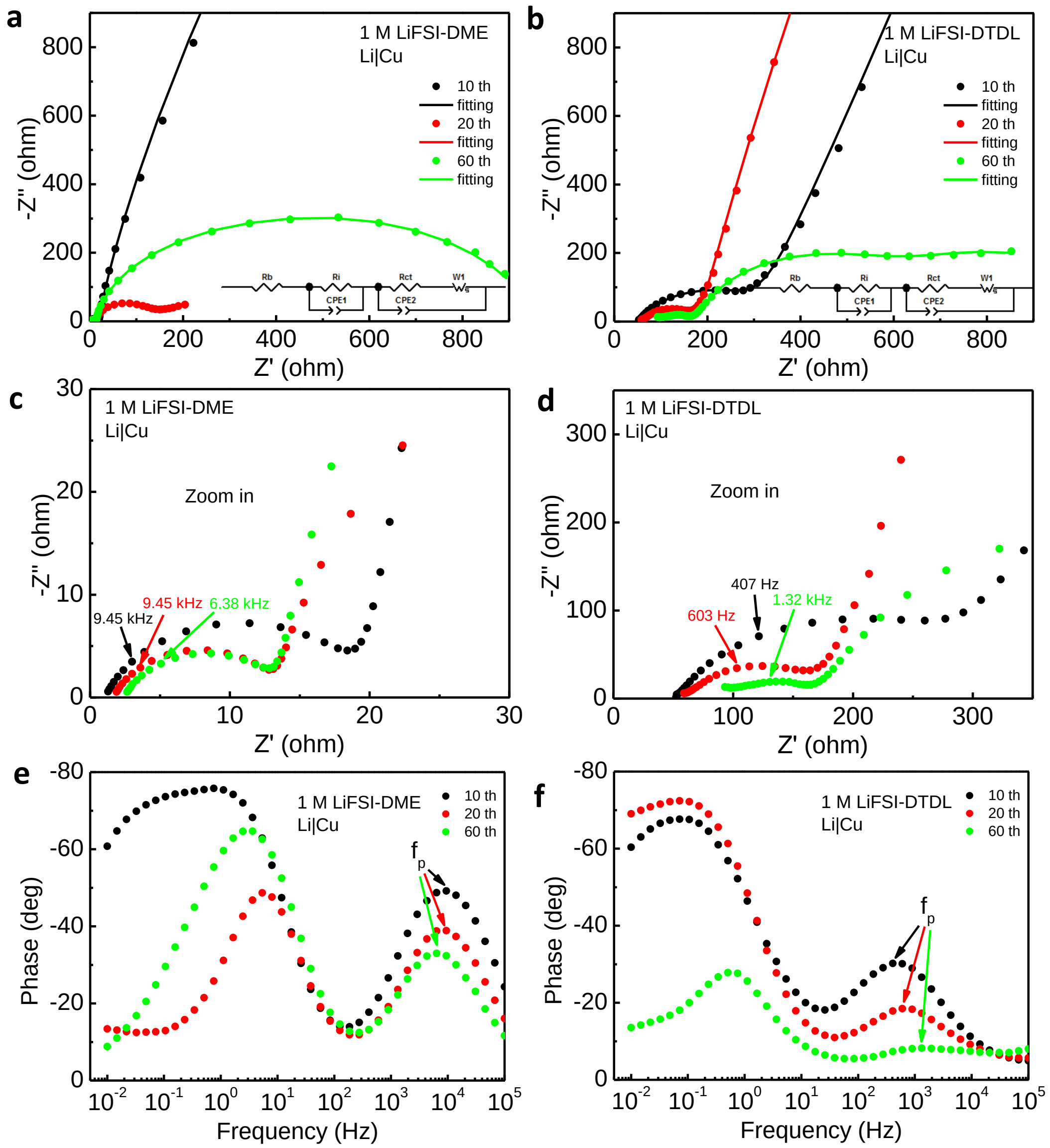

Supplementary Figure 19. EIS curves and Bode plots of Li|Cu half cells after different cycles in 1 M LiFSI-DME (a, c, e) and 1 M LiFSI-DTDL (b, d, f) electrolytes at 1 mA cm^{-2} with a cut-off capacity of 1 mAh cm^{-2}. The marked frequencies in the EIS curves are peak frequencies (f_p).

Supplementary Table 2. The EIS fitting results of Supplementary Figure 19.

	1 M LiFSI-DME			1 M LiFSI-DTDL		
Cycle No.	R_b (Ω)	R_i (Ω)	R_{ct} (Ω)	R_b (Ω)	R_i (Ω)	R_{ct} (Ω)
10 th	1.11	18.58	754.60	51.98	263.90	525.00
20 th	1.71	11.89	92.05	57.23	129.70	409.90
60 th	2.47	11.05	379.60	72.41	121.60	360.00

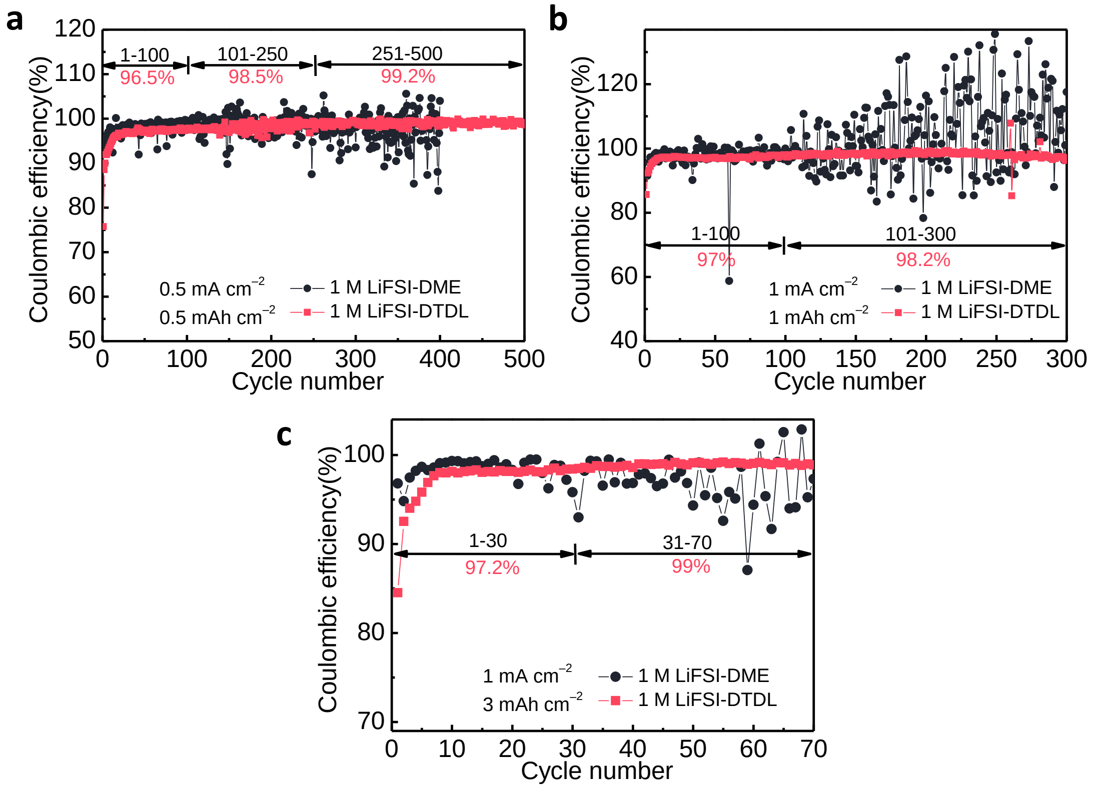

Supplementary Figure 20. Cycling test for the Li metal CE in Li|Cu half cells using different electrolytes at 0.5 mA cm^{-2} with a cut-off capacity of 0.5 mAh cm^{-2} (a), 1 mA cm^{-2} with a cutoff capacity of 1 mAh cm^{-2} (b) and 1 mA cm^{-2} with a cut-off capacity of 3 mAh cm^{-2}(c).

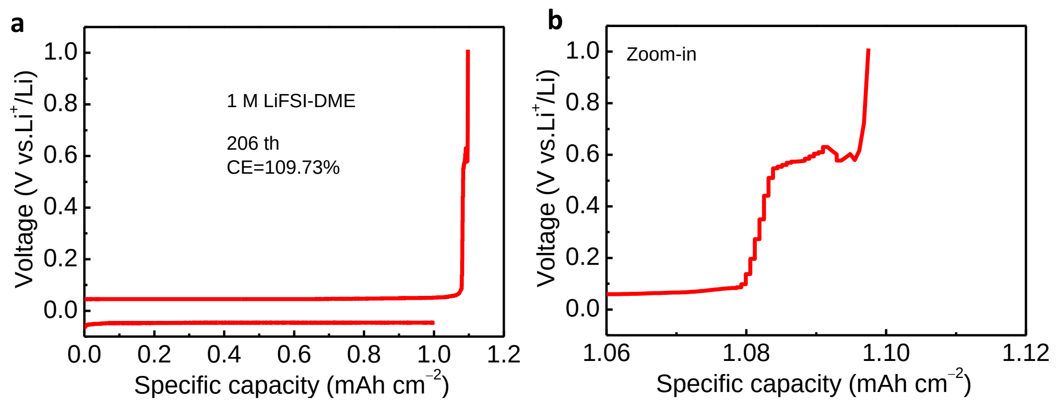

Supplementary Figure 21. Charge-discharge curve at 206th cycle in Li|Cu cell using 1 M LiFSI-DME electrolytes at 1 mA cm^{-2} with a cutoff capacity of 1 mAh cm^{-2}.

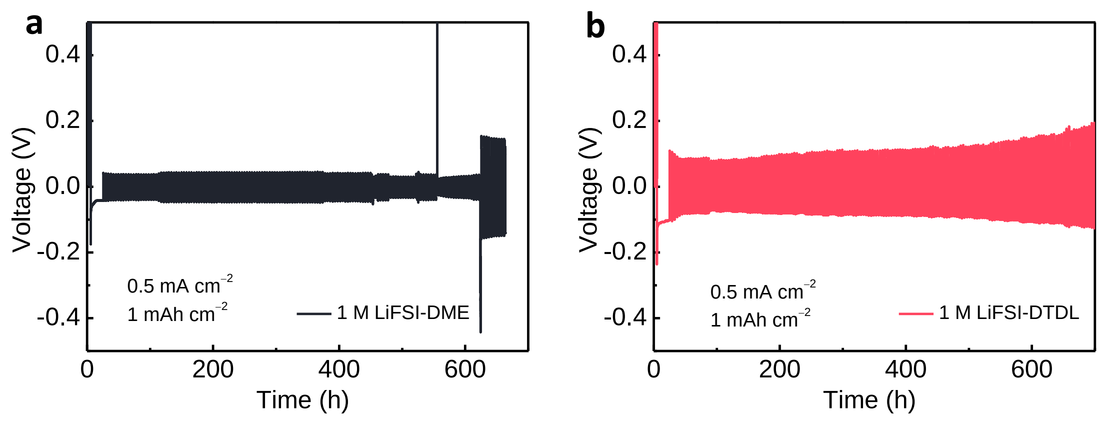

Supplementary Figure 22. Voltage−time profiles of Li|Li symmetric cells in 1 M LiFSI-DME (a) and 1 M LiFSI-DTDL (b) electrolytes at 0.5 mA cm^{-2} with a cutoff capacity of 1 mAh cm^{-2}, Li amount of 10 mAh cm^{-2} was pre-plated on Cu current collector.

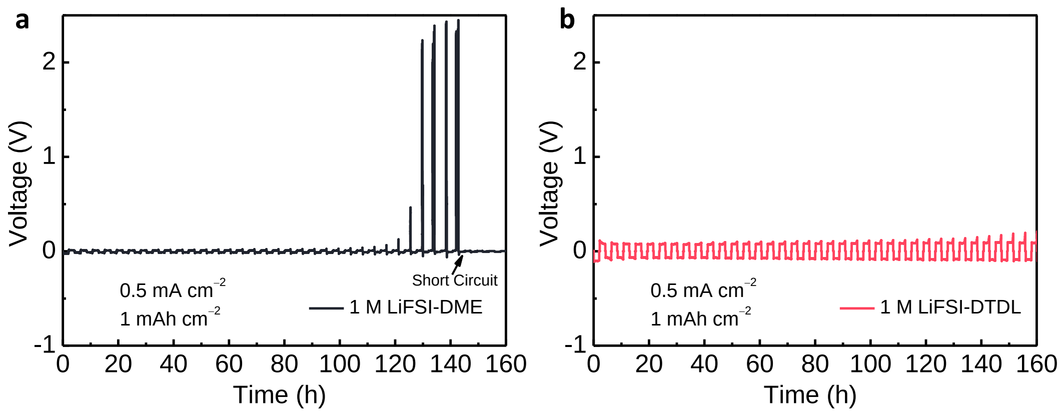

Supplementary Figure 23. Voltage−time profiles of Li|Li symmetric cells in 1 M LiFSI-DME (a) and 1 M LiFSI-DTDL (b) electrolytes at 0.5 mA cm^{-2} with a cutoff capacity of 1 mAh cm^{-2}, Li amount of 1 mAh cm^{-2} was pre-plated on Cu current collector.

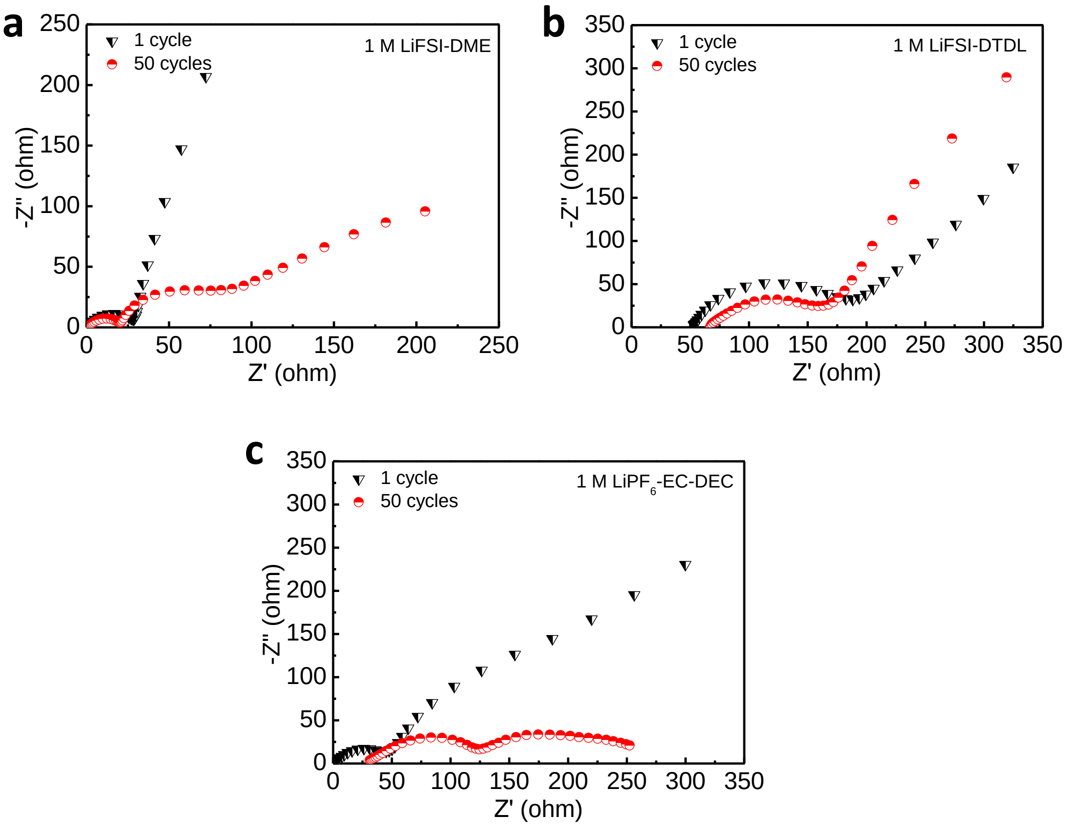

Supplementary Figure 24. EIS curves of Li|Cu half cells after first cycle and 50th cycle in 1 M LiFSI-DME (a), 1 M LiFSI-DTDL (b) and 1 M $LiPF_6$-EC-DEC (c) electrolytes at 1 mA cm^{-2} with a cut-off capacity of 3 mAh cm^{-2}.

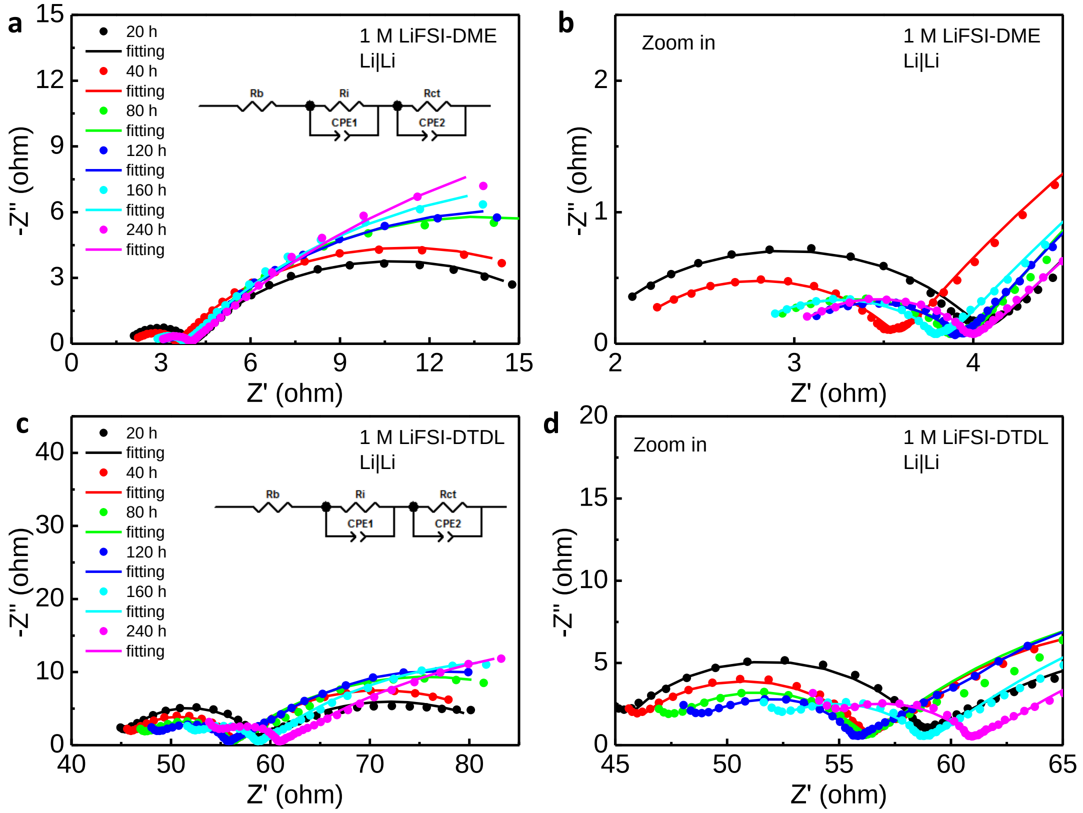

Supplementary Figure 25. EIS curves of Li|Li symmetrical cells at different cycling times in 1 M LiFSI-DME (a, b) and 1 M LiFSI-DTDL (c, d) electrolytes at 0.5 mA cm^{-2} with a cut-off capacity of 1 mAh cm^{-2}.

Supplementary Table 3. The EIS fitting results of Supplementary Figure 25.

	1 M LiFSI-DME			1 M LiFSI-DTDL		
Time	R_b (Ω)	R_i (Ω)	R_{ct} (Ω)	R_b (Ω)	R_i (Ω)	R_{ct} (Ω)
20 h	1.83	2.24	13.49	44.73	13.91	26.92
40 h	2.02	1.57	15.27	45.11	11.25	29.44
80 h	2.63	1.35	19.53	46.51	9.68	39.16
120 h	2.93	1.01	21.66	47.29	8.74	42.85
160 h	2.67	1.17	27.28	50.68	8.22	52.12
240 h	2.92	1.09	41.21	52.85	8.02	72.66

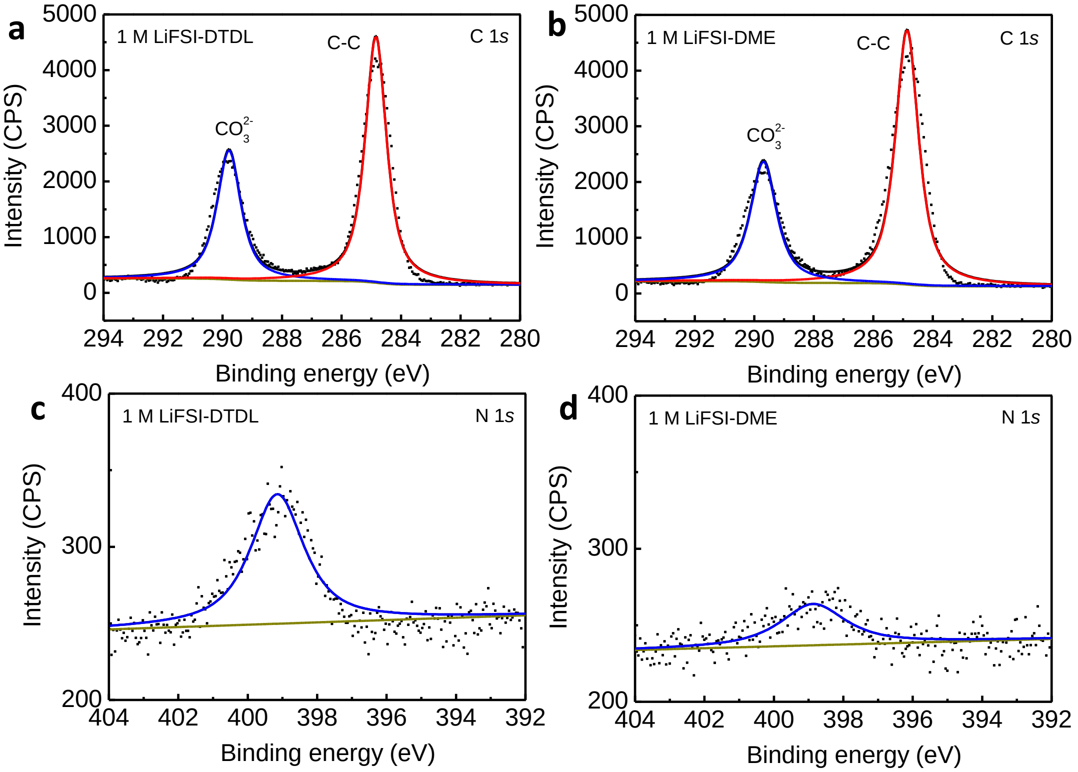

Supplementary Figure 26. C 1*s* XPS profiles on Li metal in 1 M LiFSI-DTDL (a) and 1 M LiFSI-DME (b) electrolytes. N 1*s* XPS profiles on Li metal in 1 M LiFSI-DTDL (c) and 1 M LiFSI-DME (d) electrolytes. All tests were conducted on the plated Li metal surface after 5 cycles at 1 mA cm^{-2} with a cutoff capacity of 1 mAh cm^{-2}.

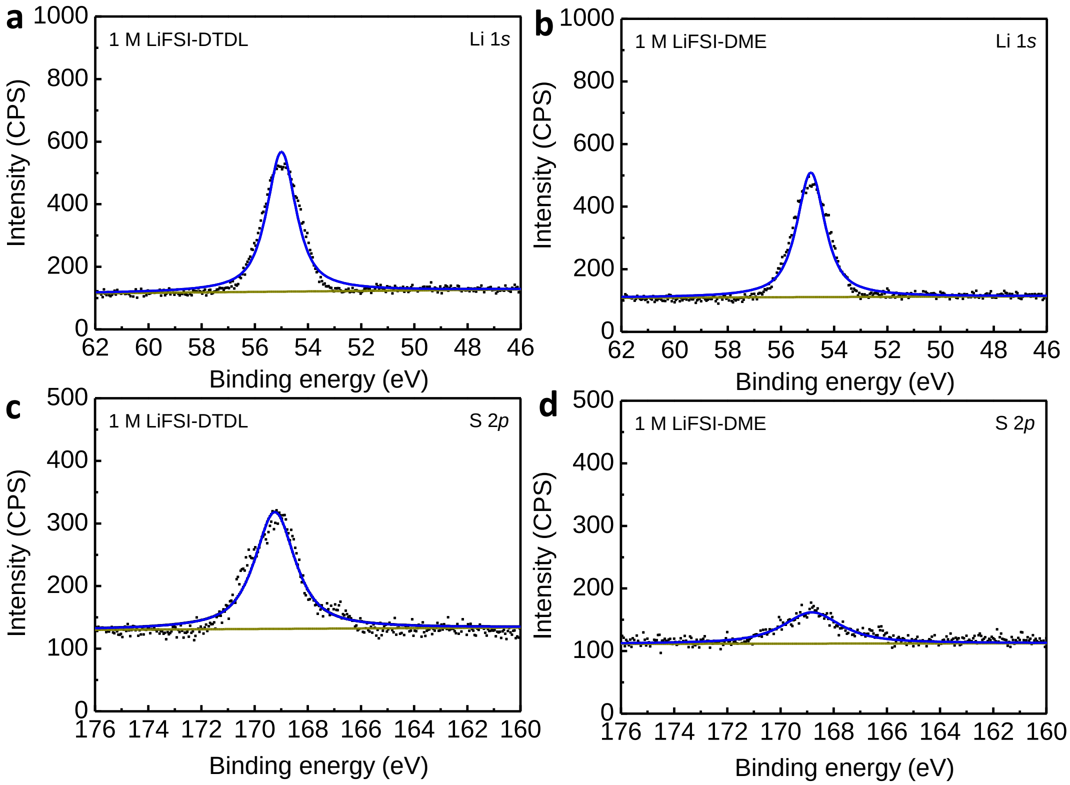

Supplementary Figure 27. Li 1*s* XPS profiles on Li metal in 1 M LiFSI-DTDL (a) and 1 M LiFSI-DME (b) electrolytes. S 2*p* XPS profiles on Li metal in 1 M LiFSI-DTDL (c) and 1 M LiFSI-DME (d) electrolytes. All tests were conducted on the plated Li metal surface after 5 cycles at 1 mA cm^{-2} with a cut-off capacity of 1 mAh cm^{-2}.

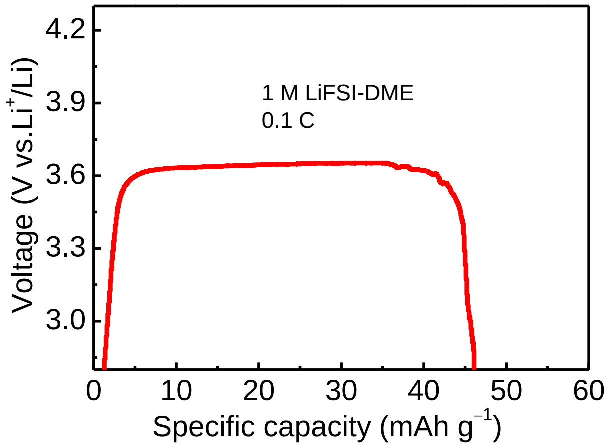

Supplementary Figure 28. Charge profile of Li|NCM811 full cells at 0.1 C in 1 M LiFSI-DME electrolyte.

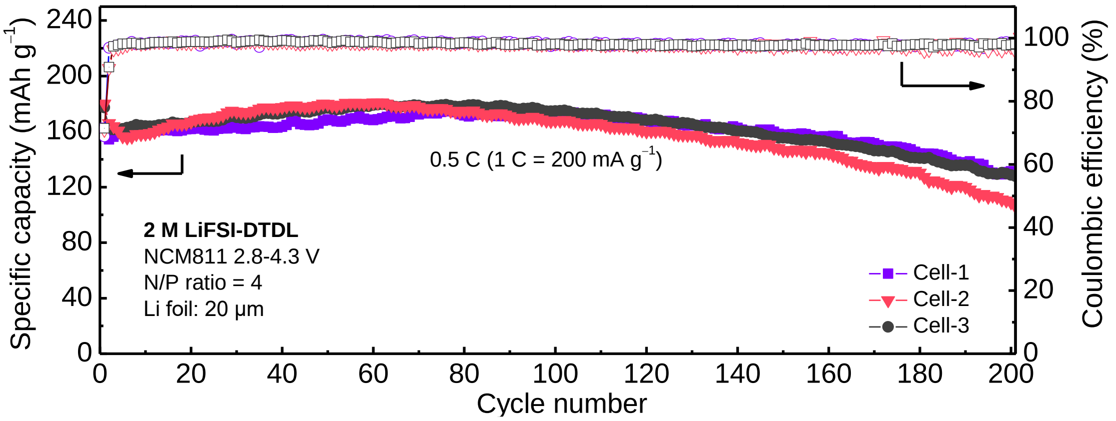

Supplementary Figure 29. Reproducibility of Li|NCM811 full cells at 0.5 C after first formation cycle at 0.1 C in 2 M LiFSI-DTDL electrolyte.

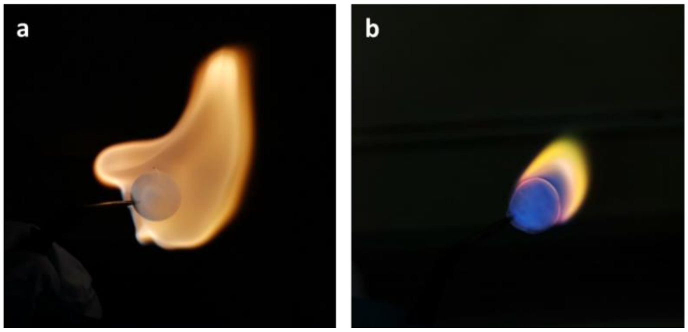

Supplementary Figure 30. Optical images of flammability tests for 1 M $LiPF_6$-EC-DEC (a) and 2 M LiFSI-DTDL (b) electrolytes.

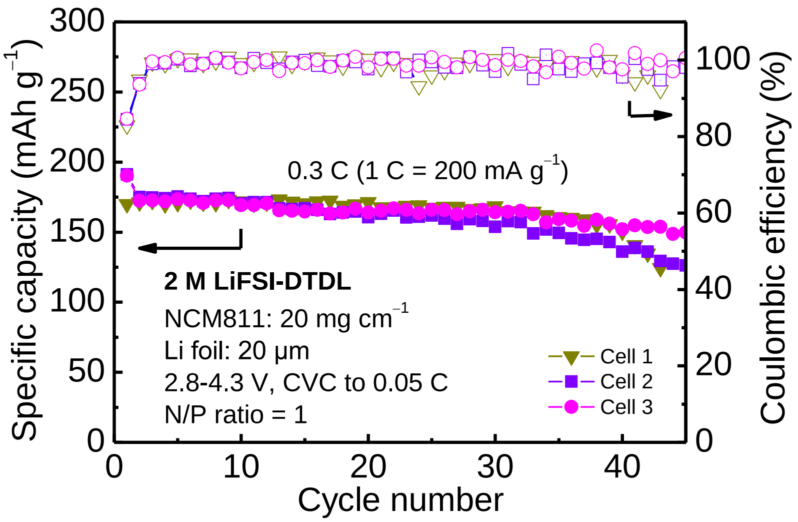

Supplementary Figure 31. Reproducibility of high loading Li|NCM811 full cells at 0.3 C after first formation cycle at 0.1 C in 2 M LiFSI-DTDL electrolyte.

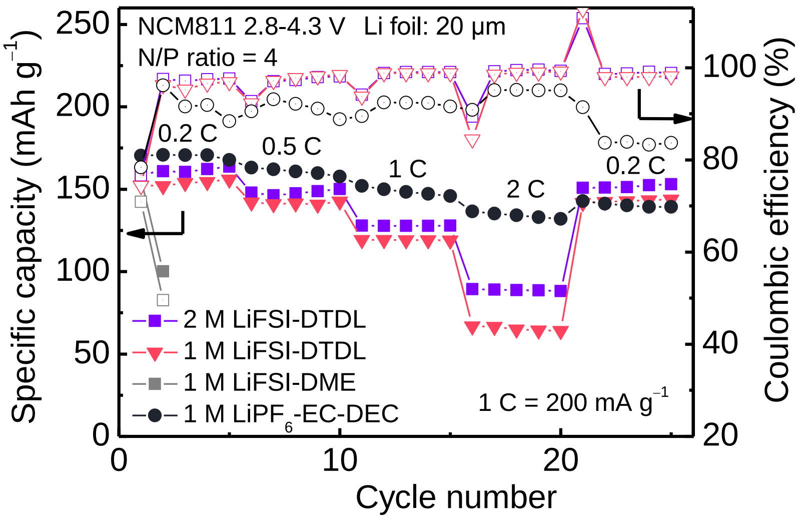

Supplementary Figure 32. Rate performance of full cells with difference electrolytes.

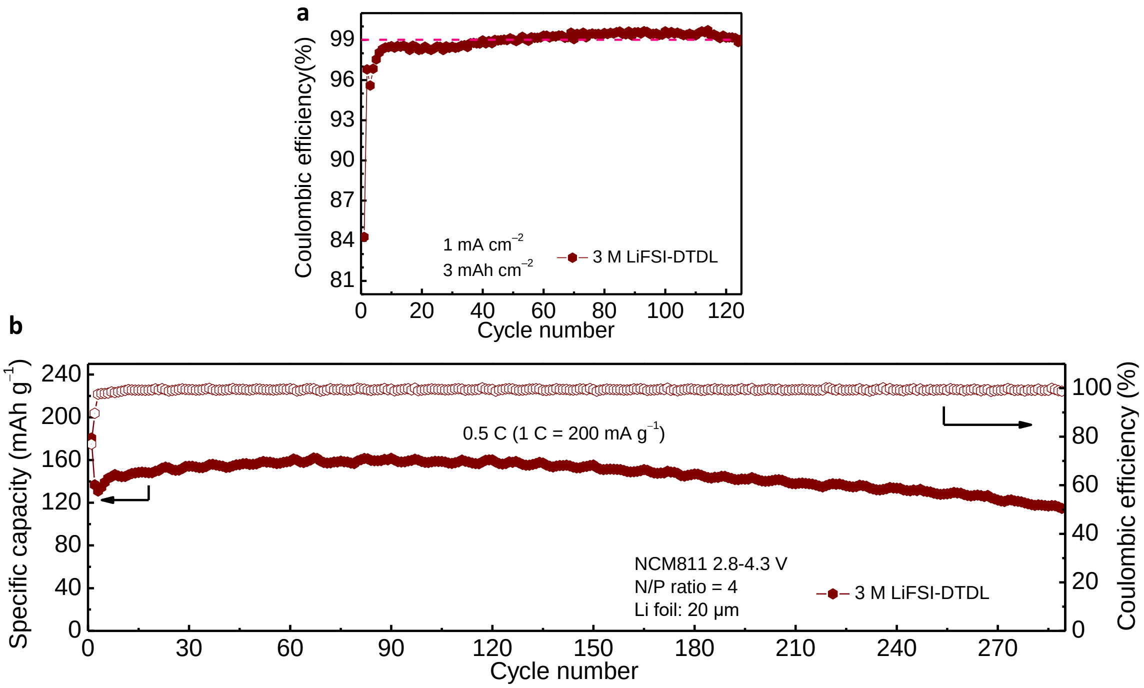

Supplementary Figure 33. Cycling stability of Li|Cu half cells using 3 M LiFSI-DTDL at 1 mA cm^{-2} with a cutoff capacity of 3 mAh cm^{-2} (a) and cycling performance of Li|NCM811 full cells at 0.5 C after the first formation cycle at 0.1 C using 3 M LiFSI-DTDL.

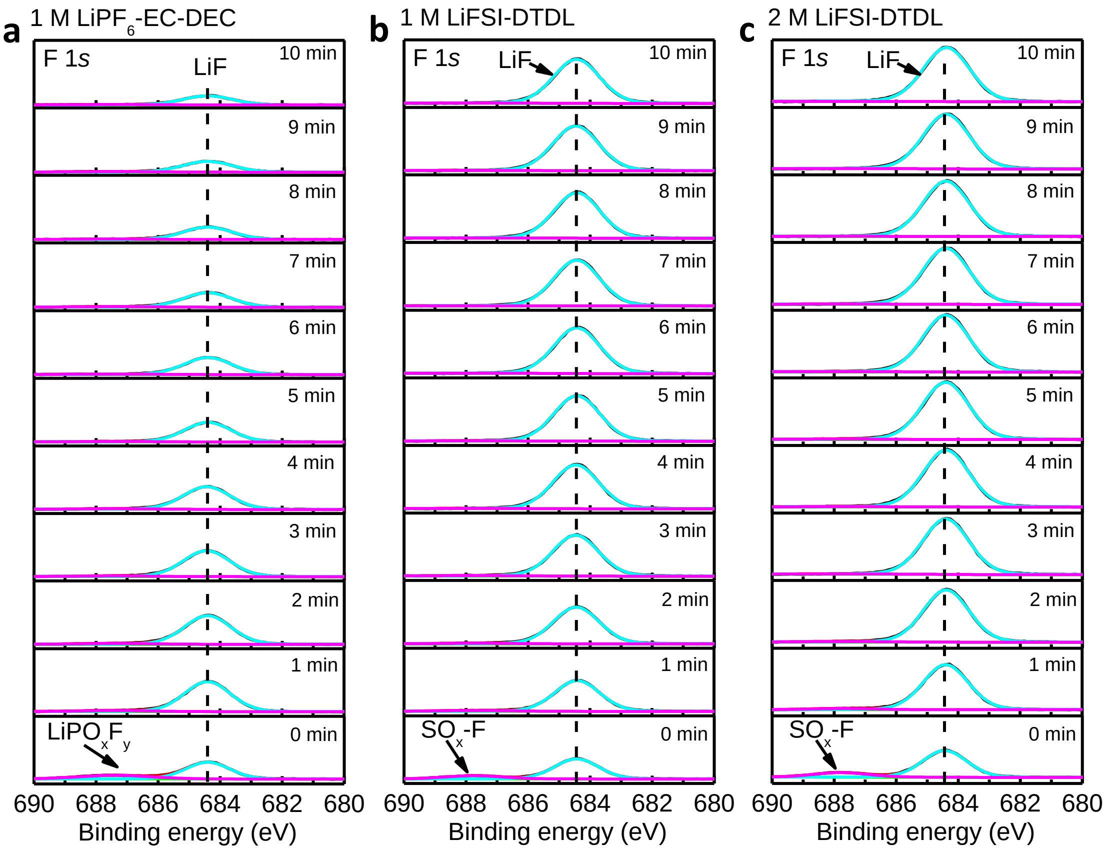

Supplementary Figure 34. F 1*s* XPS depth profiles of SEI in in Li|NCM811 full cells after 30 cycles at 0.5 C with different electrolytes.

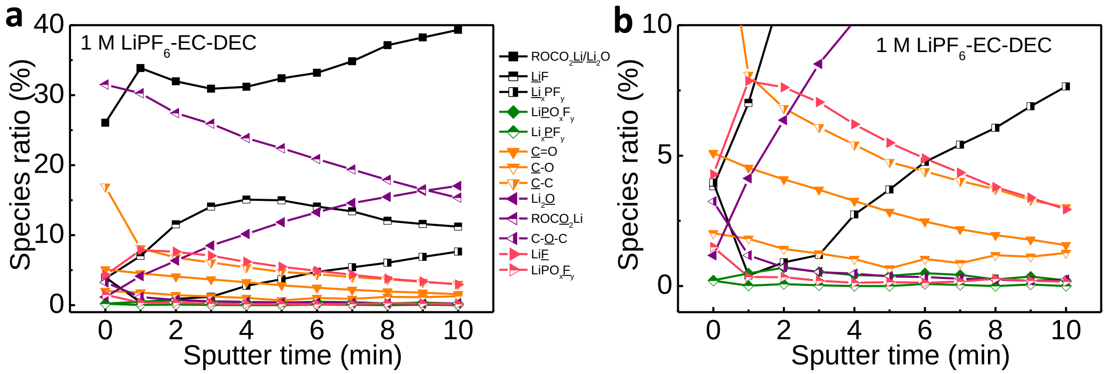

Supplementary Figure 35. The plot of quantified species ratio of SEI (a) and its zoom-in version at different sputter time in Li|NCM811 full cells after 30 cycles at 0.5 C with 1 M $LiPF_6$-EC-DEC.

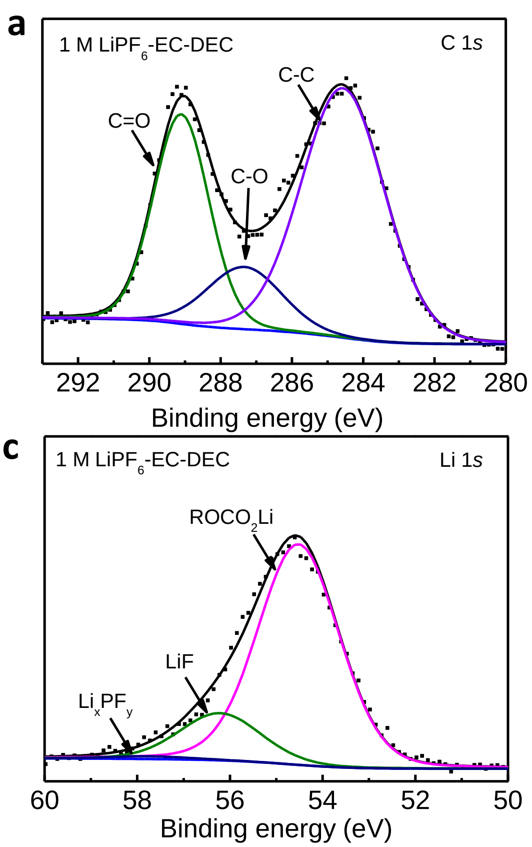

Supplementary Figure 36. C 1*s*, F 1*s*, Li 1*s* and O 1*s* spectra of SEI after sputtering 1 minute in Li|NCM811 full cells after 30 cycles at 0.5 C with 1 M $LiPF_6$-EC-DEC.

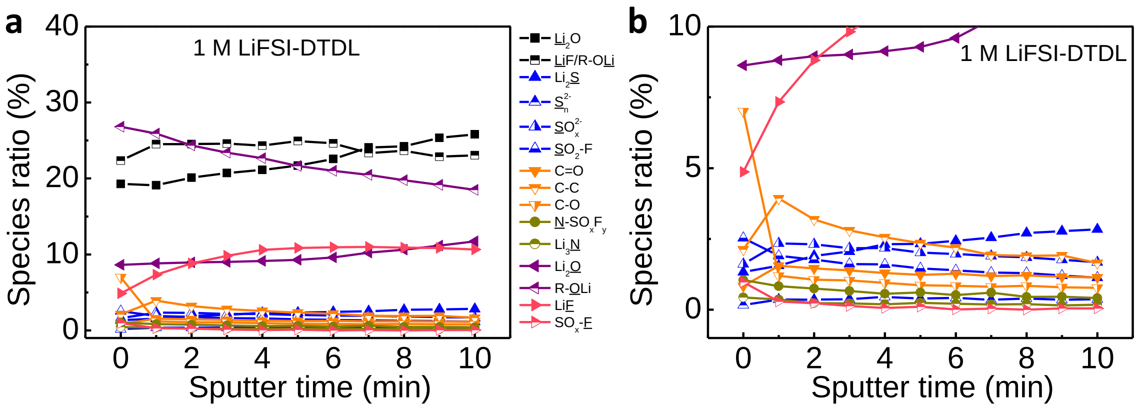

Supplementary Figure 37. The plot of quantified species ratio of SEI (a) and its zoom-in version at different sputter time in Li|NCM811 full cells after 30 cycles at 0.5 C with 1 M LiFSI-DTDL.

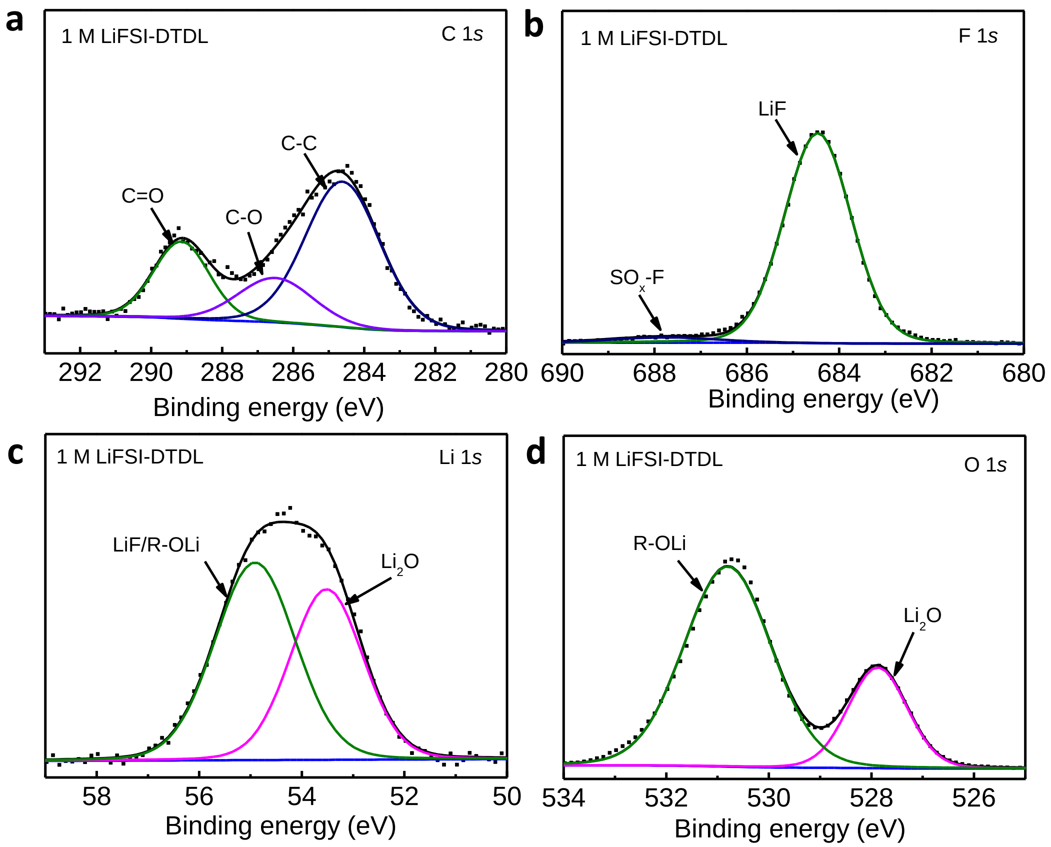

Supplementary Figure 38. C 1*s*, F 1*s*, Li 1*s* and O 1*s* spectra of SEI after sputtering 1 minute in Li|NCM811 full cells after 30 cycles at 0.5 C with 1 M LiFSI-DTDL.

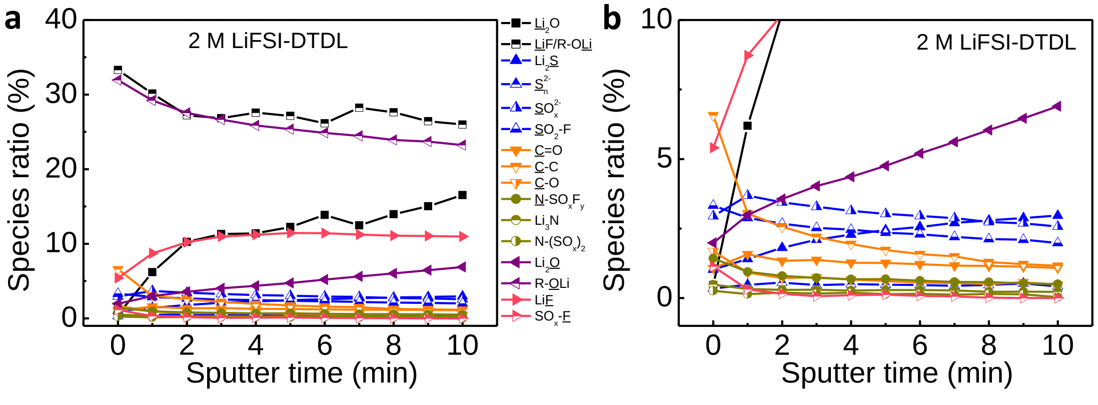

Supplementary Figure 39. The plot of quantified species ratio of SEI (a) and its zoom-in version at different sputter time in Li|NCM811 full cells after 30 cycles at 0.5 C with 2 M LiFSI-DTDL.

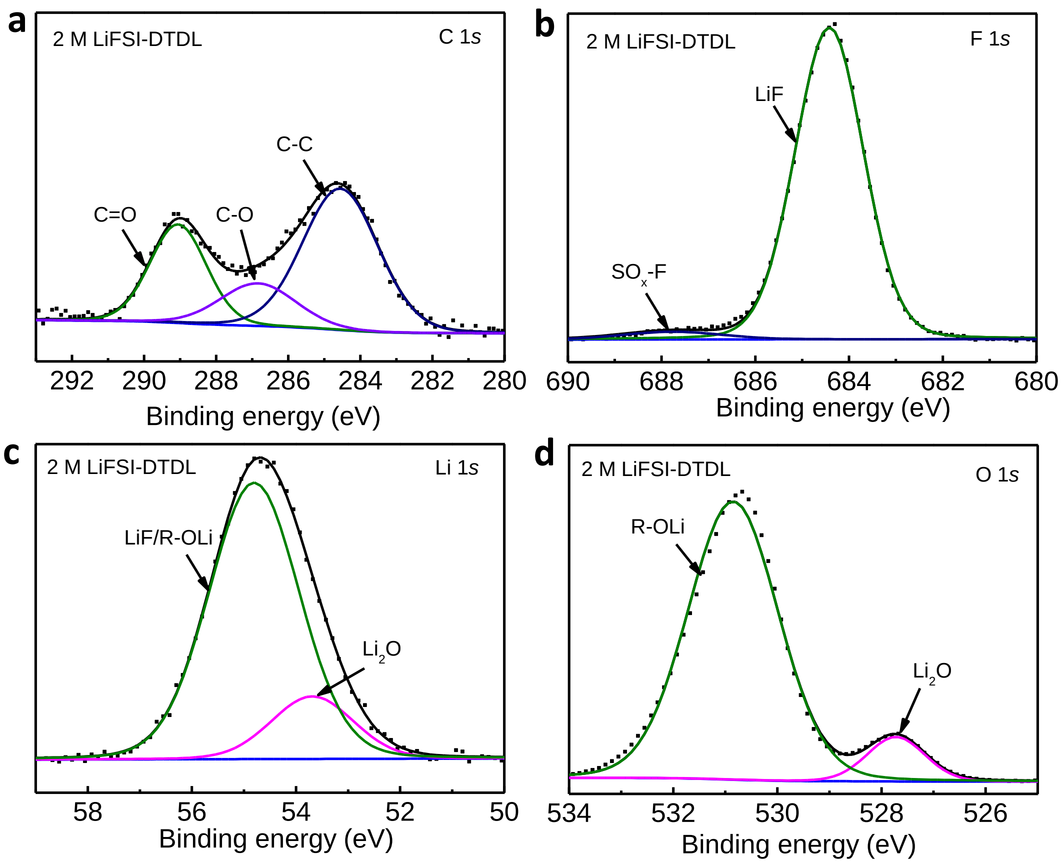

Supplementary Figure 40. C 1*s*, F 1*s*, Li 1*s* and O 1*s* spectra of SEI after sputtering 1 minute in Li|NCM811 full cells after 30 cycles at 0.5 C with 2 M LiFSI-DTDL.

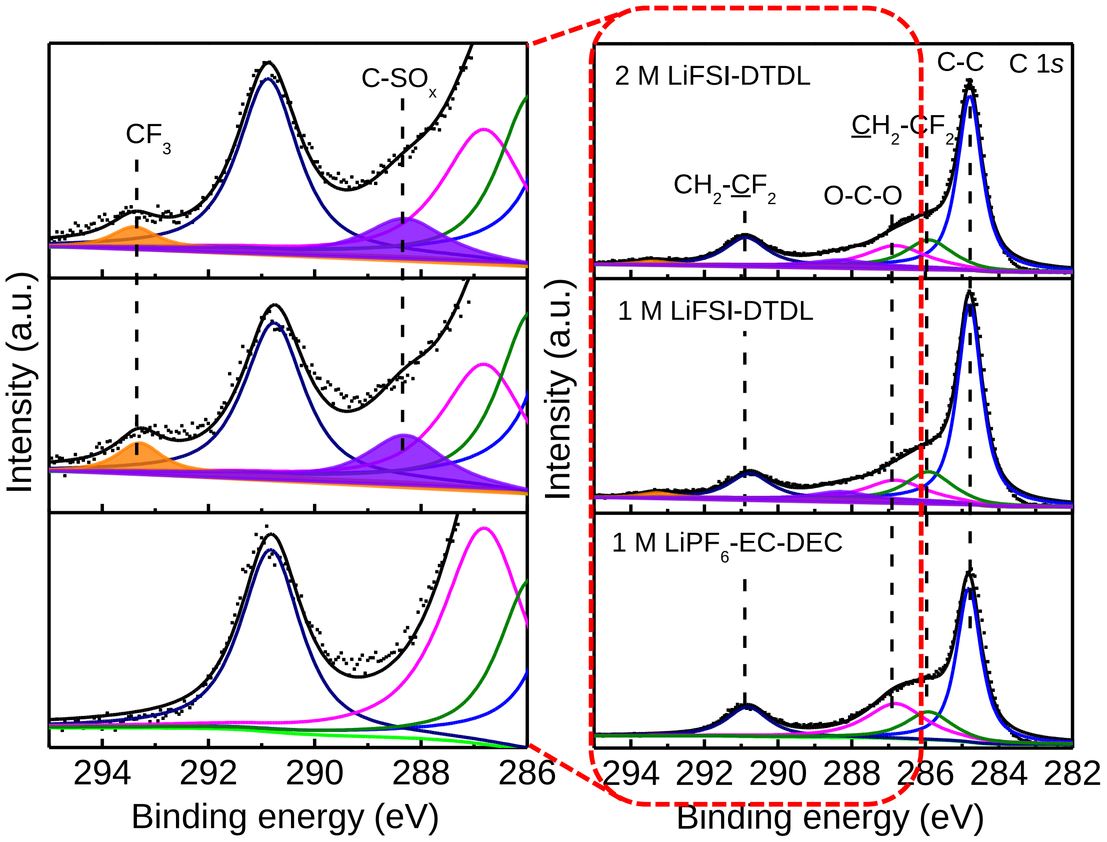

Supplementary Figure 41. C 1*s* XPS profiles on NCM811 cathode surface in Li|NCM811 full cells with different electrolytes after 30 cycles at 0.5 C.

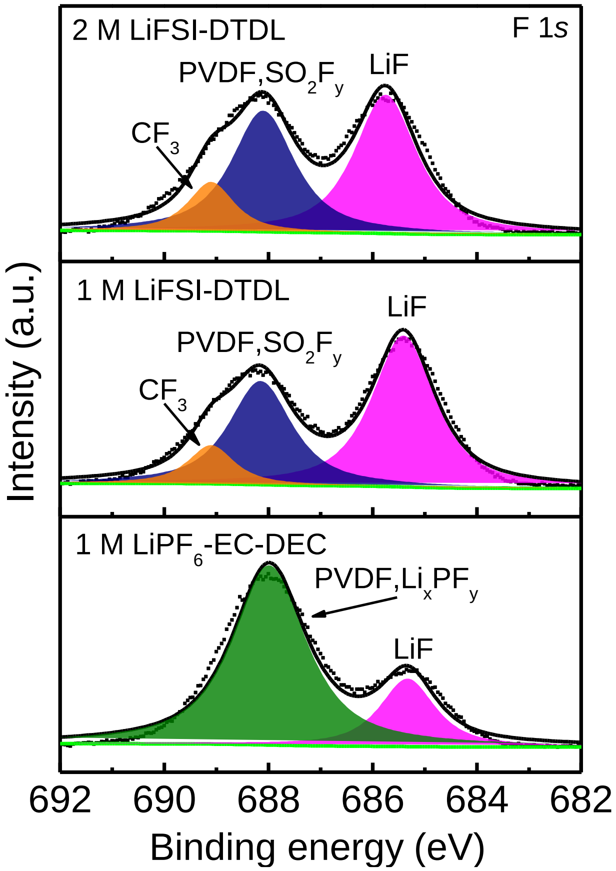

Supplementary Figure 42. F 1*s* XPS profiles on NCM811 cathode surface in Li|NCM811 full cells with different electrolytes after 30 cycles at 0.5 C.

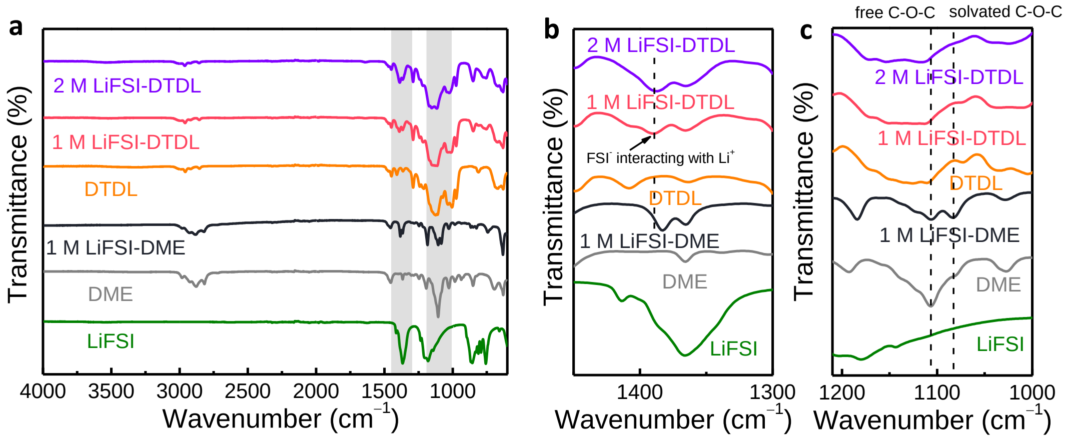

Supplementary Figure 43. FT-IR spectra of different solvents and electrolytes (a) and highlighted regions (b, c).
